# Quorum Sensing Desynchronization Leads to Bimodality and Patterned Behaviors

**DOI:** 10.1371/journal.pcbi.1004781

**Published:** 2016-04-12

**Authors:** David N. Quan, Chen-Yu Tsao, Hsuan-Chen Wu, William E. Bentley

**Affiliations:** 1 Fischell Department of Bioengineering, University of Maryland, College Park, Maryland, United States of America; 2 Institute for Bioscience and Biotechnology Research, College Park, Maryland, United States of America; Stony Brook University, UNITED STATES

## Abstract

Quorum Sensing (QS) drives coordinated phenotypic outcomes among bacterial populations. Its role in mediating infectious disease has led to the elucidation of numerous autoinducers and their corresponding QS signaling pathways. Among them, the Lsr (LuxS-regulated) QS system is conserved in scores of bacteria, and its signal molecule, autoinducer-2 (AI-2), is synthesized as a product of 1-carbon metabolism. Lsr signal transduction processes, therefore, may help organize population scale activities in numerous bacterial consortia. Conceptions of how Lsr QS organizes population scale behaviors remain limited, however. Using mathematical simulations, we examined how desynchronized Lsr QS activation, arising from cell-to-cell population heterogeneity, could lead to bimodal Lsr signaling and fractional activation. This has been previously observed experimentally. Governing these processes are an asynchronous AI-2 uptake, where positive intracellular feedback in Lsr expression is combined with negative feedback between cells. The resulting activation patterns differ from that of the more widely studied LuxIR system, the topology of which consists of only positive feedback. To elucidate differences, both QS systems were simulated in 2D, where cell populations grow and signal each other via traditional growth and diffusion equations. Our results demonstrate that the LuxIR QS system produces an ‘outward wave’ of autoinduction, and the Lsr QS system yields dispersed autoinduction from spatially-localized secretion and uptake profiles. In both cases, our simulations mirror previously demonstrated experimental results. As a whole, these models inform QS observations and synthetic biology designs.

## Author Summary

Bacterial behavior is responsive to a multitude of soluble molecular cues. Among them are self-secreted autoinducers that control quorum sensing (QS) processes. While new quorum sensing systems are constantly being discovered, several systems have been well defined in terms of their molecular and genetic topologies, each influencing a variety of resultant phenotypes. These quorum sensing systems include LuxIR homologs that use an array of species specific autoinducers and Lsr system homologs that share a single autoinducer among numerous species. Here we suggest that the regulatory topology of these two systems mark them as opposites of a sort. Whereas the LuxIR system bears a strong positive intercellular feedback mechanism, the Lsr system bears strong negative intercellular feedback. In our simulations these differences are manifested in distinct patterns of signaling. This was readily visualized in the outward spread of autogenous LuxIR expression in a growing bacterial 2D ‘colony’ whereas a dispersed activity was produced by autogenous Lsr expression in an otherwise identical colony. Here, this dispersed activity is a reflection of bimodal Lsr expression. We show that this bimodality could arise from desynchronized Lsr driven autoinducer import (intercellular negative feedback). This may have consequences on the arrangement of downstream phenotypes.

## Introduction

**Fig 1:**
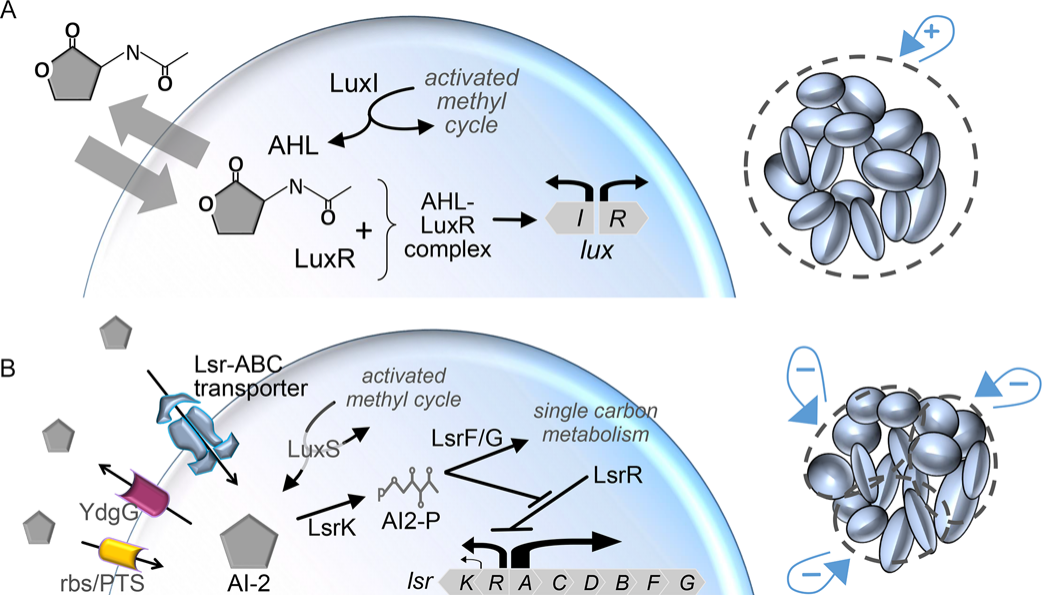
LuxIR and Lsr QS components and regulatory mechanisms. (A) The LuxIR system involves a positive feedback loop that entangles AHL synthase production with activity of the regulator LuxR. When AHL concentration is sufficiently high, LuxR is stabilized and promotes the expression of both LuxI and LuxR. As LuxI is the AHL synthase its expression completes a positive feedback loop, which is connected to *inter*cellular positive feedback through passive diffusion of AHL across the cell membrane. (B) For the Lsr system, synthesis of its autoinducer, AI-2, is produced by the activated methyl cycle and is not regulated by Lsr activity. In Lsr signaling, positive *intra*cellular feedback and negative *inter*cellular feedback arise from the same process. By the mechanisms indicated, Lsr expression stimulates a transfer of AI-2 into the intracellular space, and in doing so de-represses its own expression (positive feedback). All the while, depletion of extracellular AI-2 creates negative intercellular feedback. Important to the timing of Lsr autoinduction is the balance of AI-2 for uninduced systems. This balance is composed of AI-2 synthesis, uninduced expression of Lsr ABC-type importer, AI-2 export through YdgG, and basal AI-2 import through either ribose importers (rbs) or the PTS system.

Quorum sensing (QS) is a bacterial response to self-secreted signaling molecules known as autoinducers. While QS has been observed among individual bacteria in experimentally manipulated settings [1–3], QS often informs the coordination of processes that are metabolically burdensome and ineffectual for individual cells, yet beneficial at multicellular or population scales (e.g. virulence factor production and biofilm formation) [4]. Coordination arises from accumulated self-secreted autoinducer acting as a shared pool of extracellular signal. This regulatory strategy can focus phenotypic outcomes, reducing the effect of noise and organizing population activity [5,6]. This coordination sometimes involves the entire population such as with LuxIR QS, the signaling of which is defined by positive feedback mediated through autoinducing acylated homoserine lactones (AHL) [7] (Fig 1A). Behaviors that are driven by or occupy an entire population may not always be favored, however, as illustrated by instances of bet hedging [8] and role diversification [9]. For example, subpopulations are known to emerge in graded environments such as at the transition between biofilm margin and bulk [10,11].

The modes by which bacteria perceive and transduce the autoinducer signal can influence the extent of the QS behavior. In certain circumstances, Lsr based QS signaling via autoinducer-2 (AI-2) [12] (Fig 1B) for example, appears to generate activated and unactivated subpopulations, producing a bimodal distribution of QS activity [13,14]. Despite a fairly detailed understanding of the Lsr signal transduction process and the prevalence of Lsr in gammaproteobacteria [15,16] and AI-2 in eubacteria [17], the mechanisms underlying this coordinated fractional activation have been unexplored. Moreover, while the signaling network topology of many QS systems have been elucidated (e.g., LuxIR and Lsr systems) and mathematical models have delineated and compared a variety of QS processes [18–23], nominal consideration [24,25] has been given to Lsr QS, the extracellular negative feedback of which is unique among QS topologies.

Indeed, Lsr expression produces an AI-2 importer and kinase that work in tandem to de-repress the system, creating positive *intra*cellular feedback. This same process draws down extracellular AI-2, generating negative *inter*cellular feedback [26–29]. This is in addition to negative *intra*cellular feedback from induced LsrR and LsrFG. We hypothesized that these intertwined feedback loops could provide a rationale for observations of bimodal Lsr activation [13]. We further hypothesized that the disparate characteristics of LuxIR and Lsr activity could result in distinctive expression patterns among subpopulations. In order to explore these hypotheses, models of Lsr and LuxIR were developed and compared.

Our simulations suggest that bimodal expression of the Lsr system is a consequence of *desynchronized* Lsr activation and subsequent autoinducer recompartmentalization and catabolism, which creates a competition for autoinducer. Here, desynchronization was imposed by cell-to-cell variation of select parameters. Moreover, using a 2D finite difference agent based model of cell growth and QS signaling, we also recapitulated the expansion phase of LuxIR QS activation from a cell colony center seen in an earlier experimental study [30]. Next when LuxIR signaling mechanisms were replaced with Lsr processes, cell colonies produced dispersed pockets of Lsr autoinduction. The “speckled” expression pattern was attributed to imposed population heterogeneity. Differences in population scale signaling patterns and responses to population heterogeneity were directly related to network signaling topology.

### Model

#### Lsr QS

**Fig 2:**
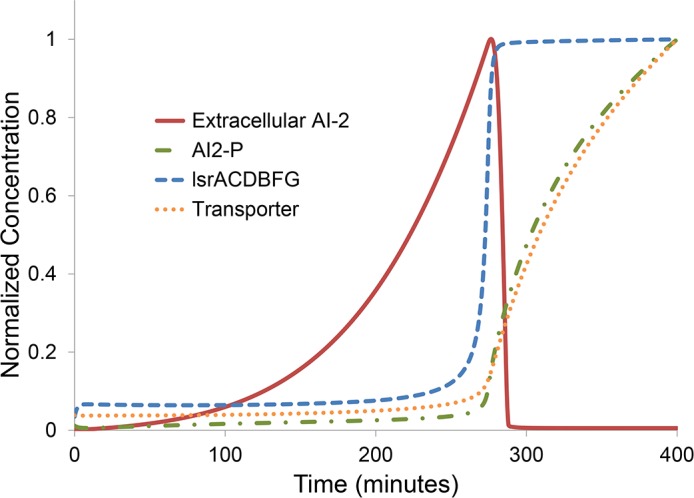
Numerical solution to system of ODEs revealing Lsr/AI-2 topology. Solution to the ODE system for select state variables representing Lsr activity. The form of ODEs is described in detail in S1 Text. As Lsr system expression dramatically increases (captured in both the increase in *lsr* mRNA transcripts (dashed line) and lagging Lsr transporter protein expression (dotted line)), extracellular AI-2 is drawn down rapidly (solid line), while intracellular AI-2 species such as phospho-AI-2 (dashed-dotted line) rapidly increase concomitantly.

Deterministic simulations were drawn from an ODE system comprised of both statistical mechanics-like and phenomenological Michaelis-Menten and Hill function terms. Parameters were chosen to be biologically realistic and to conform to Lsr expression and AI-2 activities seen in batch cultures [13,25,27,31,32]. The rationale behind the ODE design choices and parameter values are discussed further in S1 Text. Our intent was to examine topology more so than describe a specific set of experimental results. Illustrative solutions are represented in Fig 2 where shortly after 4 hours of culture Lsr mRNA expression increased abruptly while the balance of AI-2 rapidly shifted from extracellular to intracellular. Examined briefly in the S1 Fig, this system was sensitive to multiple parameters controlling AI-2 synthesis, AI-2 export, AI-2 import, AI-2 phosphorylation, and Lsr transcription. The parameter *basal* (S1E Fig) represents the rate of AI-2 import through low-affinity pathways in *E*. *coli*, possibly through ribose transporters [33] or the phosphotransferase system [34]. Along with the rate of AI-2 synthesis, baseline expression of Lsr ABC-type importer, and AI-2 export through TqsA, *basal* AI-2 import through low affinity transporters is known to play an important role in the balance of AI-2 in the uninduced Lsr system, controlling extracellular AI-2 accumulation and Lsr activation [31,33,35]. Each of these influences was represented by a separate term in the ODEs.

**Fig 3:**
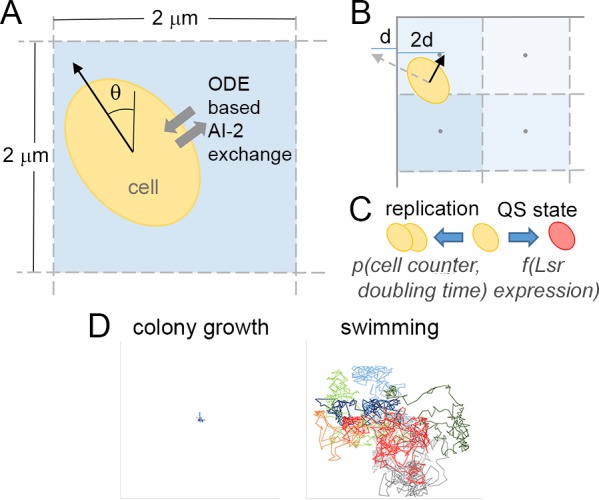
Schematic of agent based-finite difference simulation of Lsr activity. (A) Schematic of a representation of a cell within a grid element (bounded by dashed lines) moving a specific distance in a given direction (vector). The cell is exposed to the concentration of AI-2 within the grid element and conducts a net import or export according to a numerical solution for a set of ODE’s described in detail in S1 Text. (B) Multiple elements are depicted, each bearing a different shade of blue to reflect the concentration of AI-2 in each grid. Also represented is the treatment of motility at the impermeable simulation boundary (represented by solid line), where assignment of a cell motion across a boundary triggered a reflection in the opposite direction at twice the normal distance. (C) Cells also have a chance of replicating and changing their QS state in a given time step according to the values of state variables in the ODE’s. (D) Tracked motion for ten random cells from simulations of basic colony growth and unbiased movement (“swimming”) for 200 minutes after initialization. Each line represents a single cell trajectory, and each segment represents an elapsed minute. Tracks are normalized to a (0,0) starting position to elucidate patterns of motion, and due to this scale ranges from -500 to 500 μm for each axis of each plot.

This ODE model of Lsr activity was incorporated into an agent based simulation in a 500 x 500 μm 2D finite difference environment with an implied depth of 6 μm. Diagrammed in Fig 3A, space was discretized into 2x2 μm grid spaces and time into 0.0667 second steps. Also discretized was the net exchange of AI-2 between cells and the finite difference grid spaces in which they resided. Autoinducer diffusion was estimated by a central in concentration forward in time discretization, with the coefficient of diffusion estimated by the Wilke-Chang correlation [36].

Along with Lsr/AI-2 behavior, cell motility was also modeled at each time step. The distance traveled at each time step was drawn from a velocity distribution with an average of 20 μm per second and σ = 0.05 [37]. The direction (Ɵ) at each step was uniformly distributed. Movement was constrained, however, by simulation boundaries that were modeled as impermeable. Cells with projected positions outside of the simulation boundary were retained within the 2D space by relocating them at twice the normal distance away from the boundary that they otherwise would have crossed (depicted in Fig 3B). Movement was further limited such that a cell could not enter a grid with three or more cells in order to control cell density.

That is, motility within a 2D colony was first modeled entirely as a consequence of cell growth and division. For cell division events in the colony interior, one daughter cell pushed adjacent cells outward towards the closest colony edge not abutting a simulation boundary. For cells at the colony boundary, duplicated cells randomly occupied free adjacent spaces. Each time step also included the possibility of cell replication and changes in QS state, as indicated in Fig 3C. Simulations began with a cell number approximating a concentration of 0.03 OD_600_. In swimming simulations, cells were initially randomly distributed. For simulations involving colony growth, a single colony was initiated randomly in the simulated 2D space. Cell division was accounted for by converting a population-wide Monod growth rate into a per-cell doubling time that was assumed to be normally distributed from cell to cell with σ = 0.05; time to division was marked by counters for each cell. Divided cells were assigned with the same characteristics as their parent, including state variable values, QS state, etc., except for a) any parameter varied in the simulated population, b) doubling time, and c) cell position.

QS state assignment was based on the level of Lsr transporter protein. Cells were considered QS ‘positive’, ‘activated’ or ‘induced’ if the Lsr transporter protein concentration exceeded 5 times their initial concentration, and QS ‘negative’ or ‘uninduced’ otherwise. This designation was for downstream analysis and did not otherwise influence model behaviors.

Represented in Fig 3D are trajectories for ten cells from the first two hours of simulations for colony growth (left) and undirected swimming (right). As previously described, rules for motility in a 2D colony consisted entirely of movement into neighboring spaces based on cell growth events. This heuristic yielded no substantial cell trajectories. For cells following swimming motility rules, trajectories reflected the free exploration of simulated space. Such trajectories did indeed appear random, as expected.

As noted, noise or heterogeneity was applied using a Monte Carlo method to assign *basal* or (other parameter values) from lognormal distributions as a reflection of cellular constituents’ natural variability [38–41]. The assigned parameter values were maintained throughout simulated time and were considered as average values for a particular cell over that time [42,43].

#### LuxIR QS

LuxIR QS behavior was also simulated. Cells produced AHL at an average baseline rate (μ = 1 μM/min); this value was also distributed over the entire population (σ = 0.0225, log-normal distributions) to the same degree as the parameter *basal* in Lsr simulations. Once an intracellular AHL threshold of 2.9 μM was exceeded, LuxIR expression was increased linearly so that a maximal rate of AHL synthesis (10 μM/min) was obtained after a thirty minute period. This artificially captured inherent system cooperativity and the time lag associated with transcription and translation. Diffusion across the membrane was modeled by an ODE with a conductivity of 0.6 between the intracellular and extracellular spaces driven by concentration difference [19]. Cells with LuxIR QS activity were considered to be ‘active’ or ‘induced’ when AHL production was five times the basal level.

LuxIR was only simulated in the context of colony growth (no swimming). As with Lsr simulations, newly divided cells shared the same characteristics as their parent, including state variable values, QS state (‘on’ or ‘off’), etc., with the exceptions of cell position (guided by colony growth dynamics alone), the uninduced rate of AHL production (which was varied between cells), and doubling time.

## Results

### Lsr autoinduction with noise leads to fractional QS activation

In order to simulate population scale phenomena, we employed a mix of two methodologies (a comprehensive set of ODEs simulating batch cultures and a 2D agent based approach, see Model section). First, ODEs were developed to reflect the trends of Lsr activation seen experimentally in batch cultures. To this, non-genetic heterogeneity was introduced as a type of noise and source of desynchronization. Specifically, the rate of AI-2 import through low affinity transporters (described by *basal*) among the cell population was varied log normally. This created a spectrum of Lsr-activation sensitivity to AI-2 and was posited as a means of generating bimodal Lsr activity. The distribution from which *basal* values were drawn was varied in the standard deviation (σ) and mean (μ) of its natural logarithm. This was done to find the variation necessary to reflect the previously reported bimodal activation [13], but also served to explore parameters to which fractional QS activation was sensitive and to interrogate underlying system mechanics.

**Fig 4:**
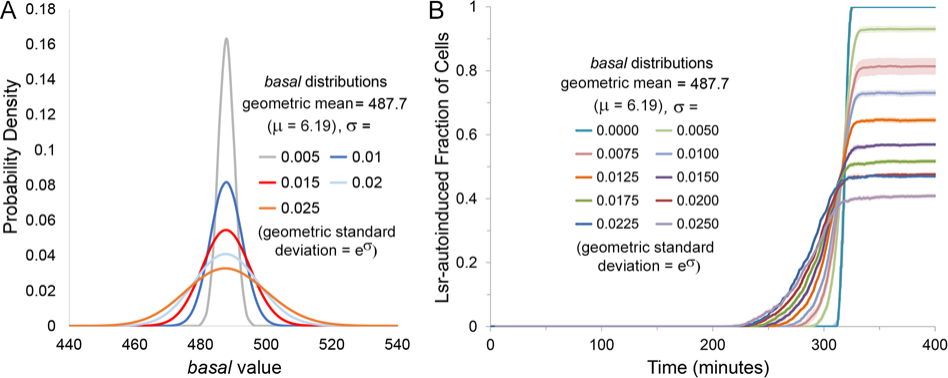
Lsr-autoinduced fraction of cell population decreases as the standard deviation of the assigned parameter *basal* increases. (A) The distribution of assigned *basal* values given select σ of the natural logarithm. (B) Results were derived from finite difference agent based simulations of Lsr activity in bacteria swimming in an unbiased manner. The fraction of the population QS activated over time was influenced by the standard deviation of the natural logarithm, σ, of the log-normal distribution for the parameter *basal*, for σ values ranging from 0 to 0.025, run in triplicate. Dark lines represent the average values, whereas lighter surrounding shade represents the standard deviation. Autoinduction initiation was slowest but most abrupt for the cell population with a standard deviation of zero, rapidly achieving activation throughout the entire cell population.

In Fig 4A we depict distributions of *basal* with different standard deviations from which parameter values were assigned. In Fig 4B, the effect of broadening *basal* distributions on Lsr autoinduction is represented. This is a plot of the fraction of activated cells over time, including the mean trajectory and its variance. The broadest *basal* distribution (σ = 0.025) produced the earliest onset of activation and the lowest final number of activated cells. A distinct trend was observed so that as the distribution became narrower, the onset of activation was delayed and a greater fraction of activated cells was obtained. Without any variation (σ = 0), Lsr QS was activated almost synchronously throughout the cell population. These results suggest that variation in *basal* coincides with and is required for the development of bimodal signaling.

**Fig 5:**
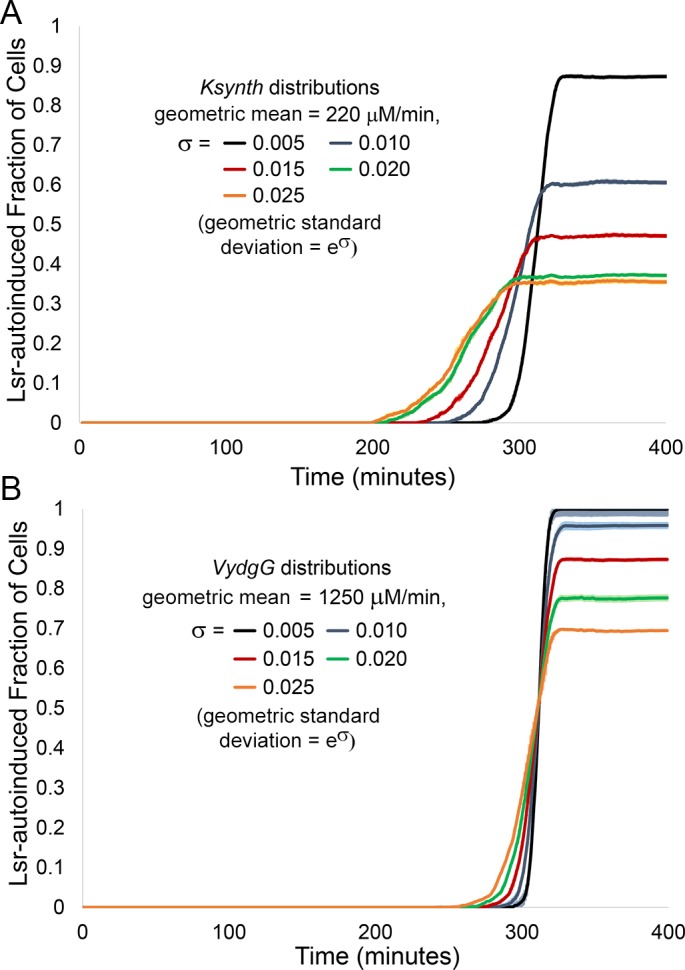
Fraction of Lsr-autoinduced cells decreases as the standard deviation of the parameters *K*_*synth*_ or *V*_*ydgG*_ increases. Results were derived from finite difference agent based simulations of Lsr activity in bacteria swimming in an unbiased manner. The influence of standard deviation of the natural logarithm, σ, of the log-normal distributions for different parameters on the fraction of the population that was Lsr-induced is represented. Standard deviation values ranging from 0.005 to 0.025 for the parameters (A) *K*_synth_ representing the rate of AI-2 production and (B) *V*_*ydgG*_ representing the rate of AI-2 export, each run in triplicate. Standard deviation of the natural logarithm for the log-normal distribution was used because the coefficient of variance is solely dependent on this measure. Dark lines represent the average values, and lighter surrounding area represents the standard deviation.

Varying the parameter *K*_*synth*_ (the rate of AI-2 synthesis) as a stand-in for cell-cell heterogeneity instead of *basal* generated similarly shifting patterns of Lsr activity. In Fig 5A, increasing standard deviations of *K*_*synth*_ (σ values ranging from 0.005 to 0.025) led to a decreasing fraction of the population ultimately becoming activated. The same range of standard deviation was also applied to the parameter *V*_*ydgG*_ (the rate of AI-2 export through YdgG). In Fig 5B, while the produced effect on fractional autoinduction was less than that for *K*_*synth*_, increasing standard deviations of *V*_*ydgG*_ led to decreasing fractions of the population becoming autoinduced nonetheless. Notably, the parameters, *K*_synth_ and *V*_*ydgG*_ affect the rate of AI-2 accumulation inversely (synthesis and export, respectively) which further supports the idea that, in general, desynchronized Lsr expression (tested here by introducing QS related heterogeneity using three independent parameters) leads to bimodal activity.

**Fig 6:**
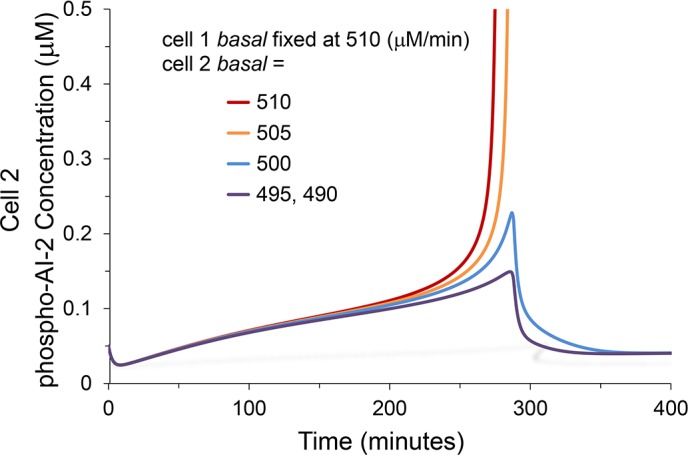
Solution to dual ODE system where *basal* of the second cell was reduced relative to the first cell. Results are from a pair of Lsr ODE’s sharing the same extracellular AI-2 but with different values for *basal*. Cell 1’s parameter values were held constant. Over separate simulations, cell 2’s parameter, *basal* (μM/min) was varied at comparatively lower values (in legend). The trajectory of AI2-P accumulation for each of these cases is represented. Rapid AI2-P increases reflects Lsr autoinduction.

Results from an ODE system modeling two Lsr networks sharing the same extracellular AI-2 pool highlighted the character of this bimodal signaling (Fig 6). The parameter *basal* of the second network was equal to or less than that of the first network, creating a difference in time to activation between the two systems. Increasing differences between the networks’ *basal* values, eventually led to the second network remaining inactive. That is, as *basal* of cell 2 decreased from that of cell 1 (510 μM/min) increases in the concentration of phospho-AI-2 in cell 2 stagnated, reflecting a two cell system where the first cell (cell 1) became autoinduced whereas the second cell (cell 2) remained uninduced.

**Fig 7:**
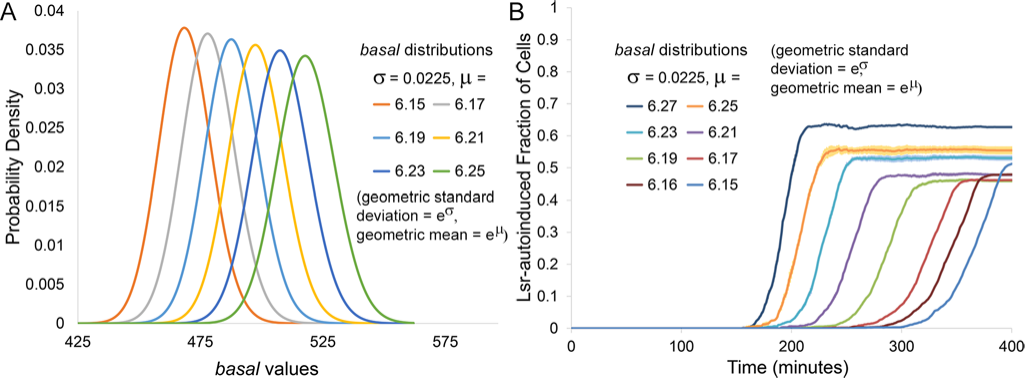
Changes in the Lsr-induced fraction of cells from changes to the *basal* mean. (A) The distribution of assigned *basal* values given select μ for the natural logarithm. (B) Presented results were derived from finite difference agent based simulations of Lsr activity in bacteria swimming in an unbiased manner. The fraction of the population QS activated over time was influenced by the mean of the natural logarithm, μ, of the log-normal distribution for the parameter *basal*, for μ values ranging from 6.15 to 6.27, run in triplicate. Dark lines represent the average values, whereas the lighter surrounding shade represents the standard deviation.

Results presented in Figs 4 through 6 suggest that the more desynchronized a population is in its time to activation, the lower the final fraction of cells expressing Lsr at high levels. For *basal* distributions in particular, the broader the variation, the earlier cells with the highest values of *basal* will begin Lsr expression and the earlier extracellular AI-2 will be depleted. Conversely, cells with lower *basal* values will thereby be increasingly prevented from autoinduction, especially as they themselves are increasingly less sensitive to AI-2. Overall, this conceptual model of desynchronized QS activation serves as a basis to explain bimodal distributions of Lsr activation [13], and further bolsters the link between increased desynchronization and lower fractional activation. The exact fraction should depend on the compound heterogeneous system characteristics that desynchronize the timing of Lsr expression. In addition to changing the variation of the *basal* distribution, its mean value was also varied (specifically the mean the natural logarithm of the distribution, Fig 7A). As seen in Fig 7B, these shifts in mean (μ = 6.17–6.27) also impacted the timing and fraction of cells that ultimately became Lsr activated. Since decreasing *basal* led to slower Lsr autoinduction in simple ODE simulations (S1 Text, S1E Fig), delayed initial Lsr expression upon decreasing the mean of the *basal* distribution was expected. The trend for the fraction of cells ultimately Lsr autoinduced was non-monotonic, however. Initially, decreasing mean and slower activation led to diminishing fractional activity. For the lowest tested means (μ = 6.15–6.17), however, further decrease and delay of initial Lsr de-repression led to slightly higher final fractional activity, producing a mixed trend, indicating that competing factors were at play in the tested range.

**Fig 8:**
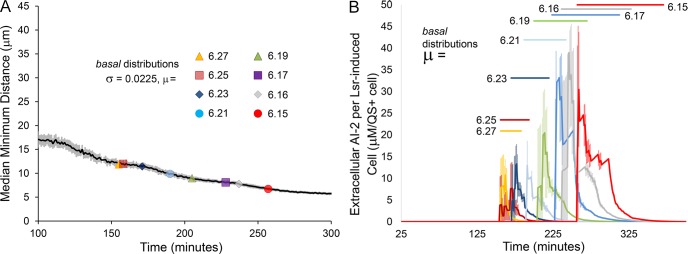
Competing influences on fractional autoinduction. (A) The median minimum cell-cell distance over time for swimming cells. The dark line is an average value (n = 20), and the surrounding lighter shade the corresponding standard deviation. Along the line are markers representing the time points at which induction began given different mean values of the distribution for the parameter *basal*. (B) The concentration of extracellular AI-2 per Lsr-induced cell from simulations using distributions of the parameter *basal* with different means. Each curve represents an average from three different simulations of swimming cells.

When starting from distributions with higher mean, decreasing quorum activation was associated with decreasing intercellular distance. Fig 8A depicts the median of nearest neighbor cell-cell distances from twenty simulations. The gradually decreasing distance resulted from growth dynamics, which are independent of Lsr activity. Markers on the line describing cell-cell distance indicate when autoinduction first begins for systems with different *basal* means, with lower *basal* means leading to later Lsr autoinduction (as described before) during periods of smaller cell-cell distances. As described in Fig 7, in tested range of *basal* means, lower values also led to decreasing fractional activation. Decreasing cell-cell distance and decreasing Lsr fractional activation could be mechanistically linked by the local diminution of AI-2 effecting more cells at these later times. This interpretation is buttressed in the Supporting Information (S1 Text and S2 Fig), where smaller integrated cell-cell distance resulting from different motility schemes led to consistently earlier initial activation and a smaller fraction of the population ultimately becoming Lsr-induced.

Any such effect was constrained, however. From previous figures, when *basal* distributions with the lower mean values were applied, we observed increasing fractional Lsr expression. Fig 8B, which represents the dynamics of extracellular AI-2 concentration per QS activated cell, provides further insight and suggests a plausible explanation. As the initiation of Lsr expression (shown by upward spikes in plot) was further and further delayed with decreasing mean *basal*, cell and extracellular AI-2 concentrations were increasingly elevated by the time Lsr-induction had begun. Under such conditions, the period between initial Lsr induction and significant reductions in extracellular AI-2 pools increased (represented by horizontal bars on plot). Effectively more cells had to become activated before AI-2 was drawn down to negligible concentrations. Put differently, as local depletion of AI-2 was delayed, more cells became Lsr-induced than would have if AI-2 depletion had been more sudden.

### Simulated Lsr and LuxIR expression in colony growth

Lsr processes were further evaluated in the context of colony growth. Most models of colony growth attempt to capture the growth and motility dynamics involved in pattern formation found on variable solid or semi-solid media substrates [44–46]. The interest here laid in the likelihood that any coordination conferred by Lsr dynamics [23] is likely to depend upon the spatial organization of the cells involved [47,48]. To avoid the need to consider complex phenotypes associated with colony growth [45] only initial colony development was modeled using space filling constraints as described previously. Both Lsr and LuxIR signaling were studied. Originally identified in *V*. *fischeri* [49], LuxIR signaling has strong positive intercellular feedback [50], and is topologically distinct from Lsr signaling. As described in the Model section, LuxIR activation was modeled heuristically, incorporating essential system features including a threshold of autoinducer required for activation, a time delay, a basal rate of autoinducer production, and a maximal rate of autoinducer production.

**Fig 9:**
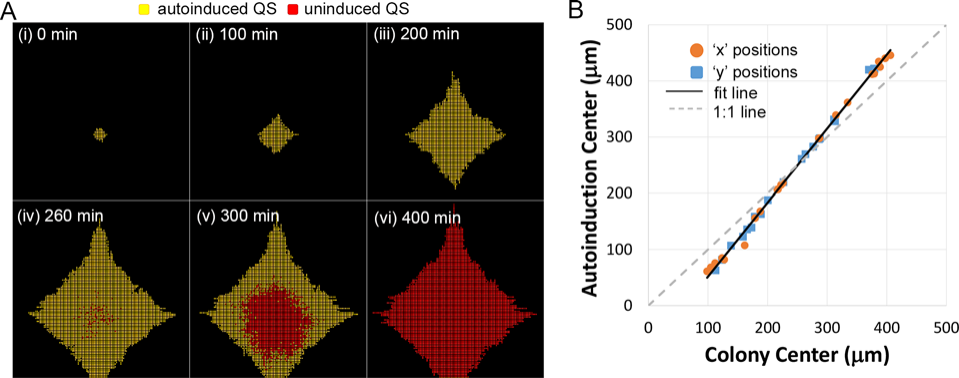
LuxIR QS dynamics coupled with gliding during colony growth generated an outward wave of autoinduction. Motility was modeled entirely as a consequence of cell growth and space filling. Daughter cells inherited state variable values from their parent cells. (A) Images from a representative simulation for LuxIR/AHL dynamics coupled with colony growth. QS active cells are in red, whereas inactive cells are in yellow. (B) When a colony center of mass was skewed toward a boundary, the center of mass for autoinduced cells was even closer to that boundary. Original ‘x’ and ‘y’ values for the colony center are mapped onto the abcissa axis, while original ‘x’ and ‘y’ values for the autoinduction center are mapped onto the ordinate axis. Original ‘x’ and ‘y’ positions from twenty simulations are marked by orange circles and blue squares, respectively. The dark line represents a linear fit to the data compared against a dashed, light gray 1:1::colony center::autoinduction center unskewed line.

A set of select images from a representative simulation for LuxIR signaling in colony growth is presented in Fig 9A. AHL concentration was initialized well below the activation threshold. Along with the selected rate of basal AHL production, this led to a couple hours delay before AHL accumulated above the QS activation threshold, accounting for the absence of QS signaling (yellow) early in initial colony growth (panels i-iii). Once initiated, however, LuxIR activity (red) spread quickly outward from its point of origin near the center of colony growth (due to positive feedback processes of cell growth and autoinduced AHL production), engulfing inactive neighbors in an outward wave of high AHL concentration, and coordinating population expression across an expanding front (panels iv-vi). The outward propagation of activity is evident in S1 Video. This activation pattern approximately recapitulated the initial expansion phase of activation seen using LuxIR engineered cells as noted above [23,30]. As in previous simulations [23,51], LuxIR initiation was biased towards simulated non-permeable boundaries. This is reported in Fig 9B, which plots positions of the colony center versus that of the initial LuxIR activation for different simulations. For each type of position, the original ‘x’ (orange squares) and ‘y’ (blue circles) are mapped onto the same axis. The simulated line deviates from 1:1 in that the simulated autoinduction consistently occurs nearer the boundary than the colony center.

**Fig 10:**
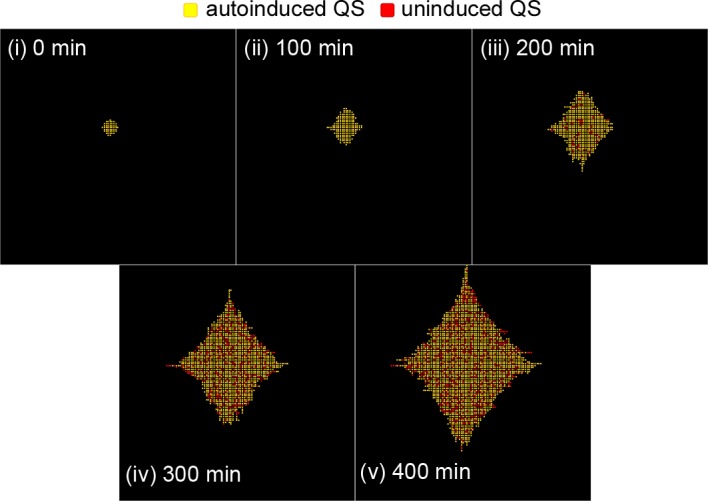
Lsr dynamics coupled with gliding during colony growth produces dispersed autoinduction. Images are from a representative simulation of Lsr induction in a growing bacterial colony. Lsr induced cells are in red, while uninduced cells are yellow. Over the course of the simulation, cells divided, duplicating the QS state and constituent concentration of their parent cells. As a result of division processes, cells pushed neighboring cells outward if necessary in the direction of the shortest path to unoccupied space.

A representative simulation of Lsr induction in colony growth is depicted in Fig 10 and S2 Video. Instead of the rapid outward spread of activity seen with LuxIR QS, after an initial delay (panels i-ii) Lsr activity (red) developed in scattered patches (panel iii). Activity remained scattered throughout the remainder of simulated colony growth (panels iv-v). Underlying this expression pattern are the dynamics governing fractional activation as described earlier. Cells moved outward to available spaces upon cell division, and produced AI-2, which accumulated fastest near the colony center. The cells most sensitive to AI-2 became Lsr-induced, leading to the uptake of nearby AI-2. Proximal cells with less sensitivity were prevented from activation. This result provides a visual depiction of the bimodal phenotype arising from Lsr based AI-2 recompartmentalization and subsequent catalysis that comprises both positive and negative feedback [26,27]. Moreover, this emphasizes the importance of spatial information in QS of all types [23,48].

**Fig 11:**
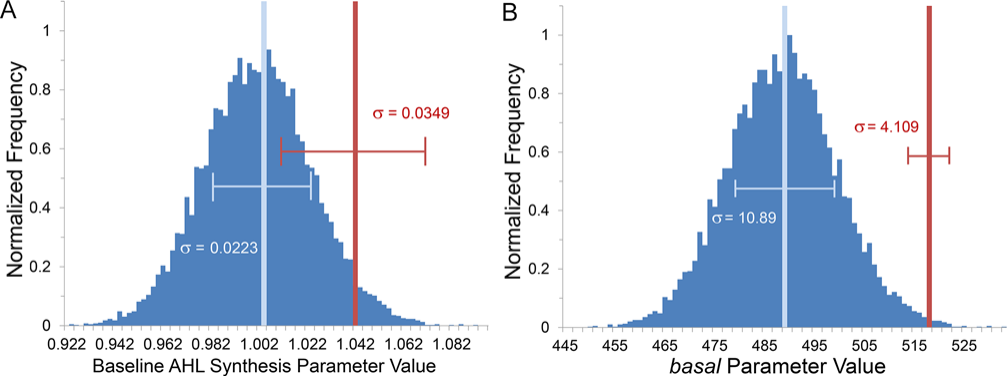
Comparison of parameter value bias among first induced cells in simulations of LuxIR and Lsr colony growth. Results from finite difference agent based simulations of LuxIR or Lsr activity in bacteria growing in a colony. Histograms of assigned parameter values (blue; event count of 10,000), with the corresponding average and standard deviation overlaid (light blue). Overlaid in red is the average parameter value of the first autoinduced cell and the corresponding standard deviation (n = 20). (A) In LuxIR simulations, where the base rate of AHL synthesis was varied, some of the first activators had parameter values less than the median indicating a smoothing effect by LuxIR on heterogeneity. (B) In Lsr simulations, where the basal rate of AI-2 import was varied, nearly all the first autoinduced cells bared the highest *basal* values.

Additional analysis attributes the differences in induction patterns to the different mechanisms by which QS systems deal with heterogeneity. This is shown in Fig 11 where the assigned values of the baseline AHL synthesis rate (LuxIR, Fig 11A) or the *basal* AI-2 uptake rate (Lsr, Fig 11B) are provided in histogram form. The vertical light blue lines represent the average and standard deviation of these rates. The vertical red lines correspond to the average and standard deviation for parameter values of the *first* cells to autoinduce. For both Lsr and LuxIR simulations the first cells to autoinduce had parameter values drawn from the higher end of each distribution. For LuxIR QS, however, the first cells to activate were not always those with the highest rate of baseline AHL synthesis, indicated by the slightly more central position of the average value along the distribution and the wider standard deviation compared to Lsr QS represented in Fig 11B. For Lsr QS, first activators had *basal* values only from the distribution tail (and carried a smaller standard deviation). In LuxIR/AHL activity the more centralized relative position and greater distribution implies that heterogeneity associated with the baseline rate of AHL production was somewhat blunted. On the other hand, the distribution of Lsr/AI-2 expression over the colony was strongly sensitive to the imposed cell heterogeneity. Quantification of the spatial heterogeneity of LuxIR and Lsr signaling in colony growth is presented in S3 Fig.

Despite the dissimilarities between colony growth and traditional experimental biofilm models [52], the development of dispersed Lsr autoinduction modeled here may nonetheless capture Lsr expression patterns in early adhering bacterial microcolonies [53]. As Lsr activity informs macrostructure development in *E*. *coli* biofilms [54] and may be important for biofilm development for other bacteria [55], whether patchiness in Lsr activity plays a role in the early development of amorphous patterning in biofilms remains of interest. These or similar simulations may be helpful in understanding Lsr signaling in biofilms, which would inherently involve spatial dynamics [56].

## Discussion

Our simulation results of bimodal autoinduction within a clonally homogenous cell population are consistent with experimental observations showing that the accessibility of QS activatable cells to autoinducer can define the fraction of the total cell population that generates QS phenotypes [13,57]. In Fig 2, we show results for solutions to an ODE system modeling Lsr signaling among cells in a batch culture. In Figs 4 and beyond (with the exception of Fig 6) we applied the same ODE systems into 2D agent based models to more readily visualize effects of cell movement and autoinducer diffusion. This was done by modeling the system as a collection of discrete cells having identical growth, AI-2 synthesis, and import parameters, while also explicitly accounting for diffusion and motility. Using these agent based models in Figs 4, 5 and 7 we demonstrated that heterogeneity within an otherwise clonal population, embodied here by a distribution in the basal AI-2 import rate among cells, led to a subpopulation of cells taking in AI-2 and conducting QS activity while other cells remained unactivated. The net result was fractional Lsr autoinduction at the population scale.

We note that bimodal expression arising from clonal origins is a common phenomenon, sometimes associated with pattern formation and differentiation in multicellular organisms [58,59], arising as a consequence of nonlinear responses to heterogeneity or asymmetry [60,61]. For the Lsr system, results indicate that bimodal expression arises when the intercellular negative feedback associated with Lsr activated AI-2 uptake and catabolism is desynchronized across a population (here represented by varied rates of AI-2 uptake and subsequent Lsr mediated gene expression).

Such bimodal expression may represent role diversification within the population. Bacterial diversification is frequently framed within the context of bet hedging [8] or as a graded response to environmental conditions, an example being the different conditions at the margins of a biofilm compared to those within the bulk [10]. While Lsr signaling does influence biofilm development in *E*. *coli* [62], whether population diversification through Lsr QS represents bet-hedging or specialization within the context of a population-wide transition to a sessile lifestyle remains of interest. As Lsr homologs are found in numerous bacteria [15], the heterogeneous influence of Lsr and Lsr-related systems on emergent phenotypes is likely to vary from species to species, and may only be effective in specific settings and circumstances.

By transitioning simulations to a 2D agent based model, the consequence of differences between Lsr and LuxIR mediated QS activation were made clear (Figs 9–11). For LuxIR signaling in colony growth simulations, the colony expanded its footprint as cells doubled and at the same time produced AHL that promoted increased AHL synthesis via LuxIR expression. The net phenotype was a sudden outward expansion of LuxIR expression initiated in colony centers, the regions of longest cell residence and highest accumulation of autoinducer. Instead, as governed by an autoinducer requiring active transport, Lsr system activity generated dispersed autoinduction, creating patterns similar to that of phase repulsive coupling in epilectic astrocyte cultures [63]. These distinctive spatiotemporal patterns are attributed to intracellular positive feedback processes also producing intercellular negative feedback in Lsr signaling, whereas LuxIR QS dynamics generate only positive feedback (recall Fig 1). Overall, while autoinducer clearly has a coordinating and organizing effect on cell and Lsr activity, autoinduced Lsr expression is only experienced by a fraction of the cell population, and in a manner that appears to favor dispersion of that expression in space.

Ancillary interpretations also stem from our results; though speculative, they are worthy of discussion. For instance, since desynchronization appears to result in bimodal signaling due to induced AI-2 import and catalysis, the phenomenon would seem to be sensitive to the induced rate of AI-2 import, with slower import decreasing negative intercellular feedback. This intuition bears out insofar as when the induced rate of AI-2 import is lower a greater desynchronization (via increased *basal* heterogeneity) is required to achieve fractional Lsr induction. In all other simulations presented here, the induced rate was orders of magnitude greater than the basal rate. Using an induced rate less than 6 times the basal rate (by altering one parameter), significantly greater *basal* heterogeneity was required to produce a modest non-activating population (S4 Fig). Interestingly, heterogeneity for a glucose importer was reported by FACS to be of the same order of magnitude [39]. Moreover, tracking of heterogeneous expression within a population reveals that for the bulk of cells expression levels relative to the overall distribution are qualitatively stable over the time scales modeled here [42], with the exception of outlying high or low expressers which may be possibly attributable to regulatory mechanisms [40]. Moreover, while this study simplistically focuses on the heterogeneity of a single molecular species, the cells are composed of numerous components. We have not explored whether heterogeneity associated with multiple components produces different results.

Among other larger ideas, that bimodal expression evaluated here arises from a mechanism creating both positive intracellular and negative intercellular feedback indicates that Lsr expression may represent an exception or twist to the idea that bimodality is primarily a consequence of positive feedback regulation [60,64,65]. That is, while positive feedback clearly remains an integral feature of Lsr activity, this study may be seen to suggest that in examining conditions for bimodality, extracellular interactions should be considered for cases where inducer depletion results from induction, as nontrivial patterns may arise [63].

Finally, as previously mentioned, Lsr system homologs are plentiful among gammaproteobacteria, while AI-2 production occurs among eubacteria more broadly. That being the case, results here lend support the idea that in some circumstances the Lsr system could represent an intraspecies quorum sensing mechanism that uses a “universal” signaling molecule. This would seem to have ramifications for QS cheating and competition more broadly, which this model could be used to interrogate in future studies.

## Supporting Information

S1 Fig System sensitivity to parameter changes that manifested in shifting time to Lsr autoinduction. Solutions to ODE’s modeling Lsr activity, where rapidly increasing phosphorylated AI-2 concentration indicates Lsr autoinduction. Shifts in the time to activation were associated with reported parameter value changes. Changes to *k2* (transcription Hill parameter; A), *k*_*phos*_ (phosphorylation; B), *K*_*synth*_ (AI-2 synthesis; C), *V*_*ydgG*_ (AI-2 export; D), and *basal* (low affinity AI-2 import; E) are presented.
(TIF)

S2 Fig Measures of the difference between different modes of motility when coupled with Lsr/AI-2 dynamics. Results were derived from finite difference agent based simulations of Lsr activity. (A) The fraction of the population that was QS activated over time, in Lsr simulations of different motility, with average values set in the darker lines and the standard deviation represented by lighter surrounding shades (n = 20). (B) The median minimum cell-cell distance for populations influenced by different combinations of motility and AI-2 uptake. Dark lines are average value and the surrounding lighter shades reflect the corresponding standard deviation (n = 20). For example, cells undergoing colony growth had a predefined, regular distance between them, thus a single value prevailed across the entire time course and variability was zero.
(TIF)

S3 Fig Local heterogeneity of Lsr versus LuxIR QS activation in colony growth. Results were derived from finite difference agent based simulations of LuxIR activity or Lsr activity using a median *basal* of 487.8 and a coefficient of variance of 0.052 (σ = 0.0225) for bacteria growing in a colony. (A) The dark lines represent the average local heterogeneity of 20 simulations, while the lighter, surrounding shades represent the standard deviation of those values. Also noted are the percentage of QS activity at the plateau of heterogeneity for Lsr simulation (represented in blue) and the percentage QS activity at the peak of heterogeneity for LuxIR simulations (represented in red). This is relevant, since the measure of local heterogeneity used is sensitive to the fraction of QS activation. This is seen in (B) for measures of colonies wherein QS state was assigned for each cell with a probability reflecting the percent QS activation. For enhanced context, the blue dot represents the heterogeneity for Lsr at plateau, whereas the red dot represents the heterogeneity for LuxIR at 50% activation.
(TIF)

S4 Fig Slower induced import results in less negative intercellular feedback and fewer induced cells. Results were drawn from finite difference agent based simulations of Lsr activity using a reduced rate of induced AI-2 import. The fraction of the population that was QS activated over time given changes to the variation for the distribution of the basal rate of AI-2 import. Variation was shifted over a range from 0.0 to 0.35.
(TIF)

S5 Fig Comparison of solution for population with a single basal value versus a population with a unimodally distributed value of *basal*. Juxtaposition of the solution for extracellular AI-2 for a simulation of cells with a single basal value versus the average solution of extracellular AI-2 for a simulation of cells with a log normal distribution of the parameter *basal*
(TIF)

S6 Fig Comparison of results from single versus multiple finite difference elements to define environment. The average trajectory of AI2-P for cells with the same parameter sets in simulations where the environment was defined as either a single finite difference element or by the standard array of elements as defined in the methods. Modeling with a single finite difference element eliminates spatial noise as a source of difference between cells. The addition of noise through the full implementation of finite difference elements, adds spatially associated noise to the simulation. This did not result in a significant change in the average trajectory of AI2-P.
(TIF)

S7 Fig Congruence of solution from finite difference-agent based modeling versus implicit solution of pure ODE’s. **A** AI2-P trajectory from implicit numerical methods and the average AI2-P concentration from the finite difference-agent based approach. Here, cells from the finite-difference-agent based solution all held the same parameter values as that from the pure ODE solution. In the pure ODE approach, cells were modeled as a dependent variable. Ideally, the two solutions would bear identical traces. **B** The rate to activation was assessed by fitting the function, *f(t)*, from 12–152 minutes to a first order linear regression, *g(t)*. The first time point at which *f(t)-g(t)>2g(t)* was considered the point of activation. The time to activation for each value of *basal* was calculated and the bearing on the solution by the modeling and numerical method used was evaluated by direct comparison along the primary axis and according to the ratio of activation times for the finite difference-agent based solution to the pure ODE solution on the secondary axis.
(TIF)

S1 Figure Source File Excel file of data used for figures. (XLSX)

S1 Table Table of parameters and parameter values. (DOCX)

S1 Text Supporting information text. (DOCX)

S1 Video Video of LuxIR QS dynamics coupled with gliding during colony growth. Video of a representative simulation for LuxIR/AHL dynamics coupled with colony growth. QS active cells are in red, whereas inactive cells are in yellow. The complete simulated time of the video is 400 minutes.
(MP4)

S2 Video Video of Lsr QS dynamics coupled with gliding during colony growth. Video of a representative simulation for Lsr/AI-2 dynamics coupled with colony growth. QS active cells are in red, whereas inactive cells are in yellow. The complete simulated time of the video is 400 minutes.
(MP4)

S1 XML File SBML format XML file of Lsr system model. (XML)
