# Supplementary figures and images for: Quorum Sensing Desynchronization Leads to Bimodality and Patterned Behaviors
Source: PLoS Comput Biol. 2016 Apr 12;12(4):e1004781. doi: 10.1371/journal.pcbi.1004781 (PMC4829230; doi:10.1371/journal.pcbi.1004781)

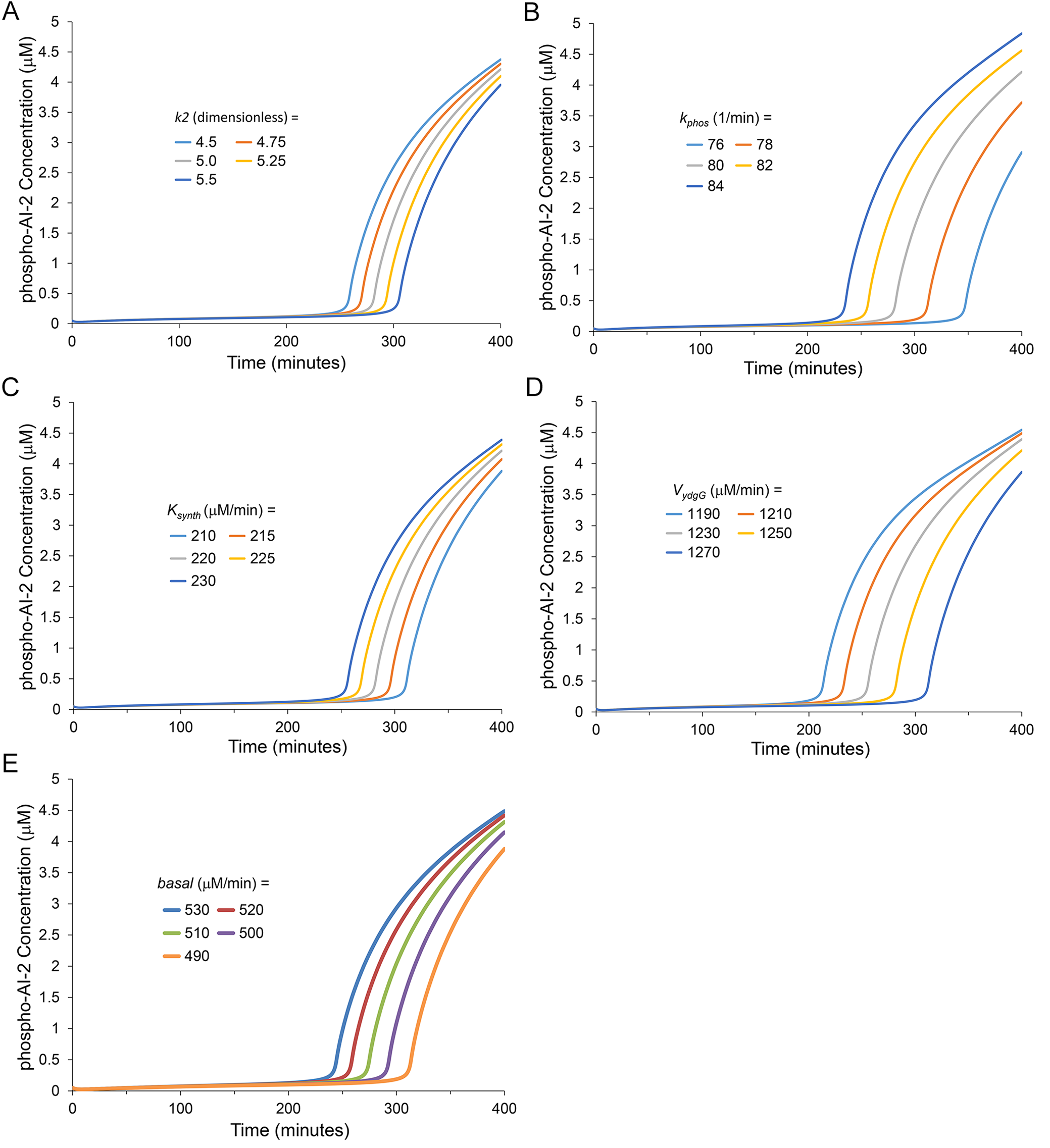

Supplement: S1 Fig — Solutions to ODE’s modeling Lsr activity, where rapidly increasing phosphorylated AI-2 concentration indicates Lsr autoinduction. Shifts in the time to activation were associated with reported parameter value changes. Changes to k2 (transcription Hill parameter; A), kphos (phosphorylation; B), Ksynth (AI-2 synthesis; C), VydgG (AI-2 export; D), and basal (low affinity AI-2 import; E) are presented. (TIF) [file pcbi.1004781.s001.tif]

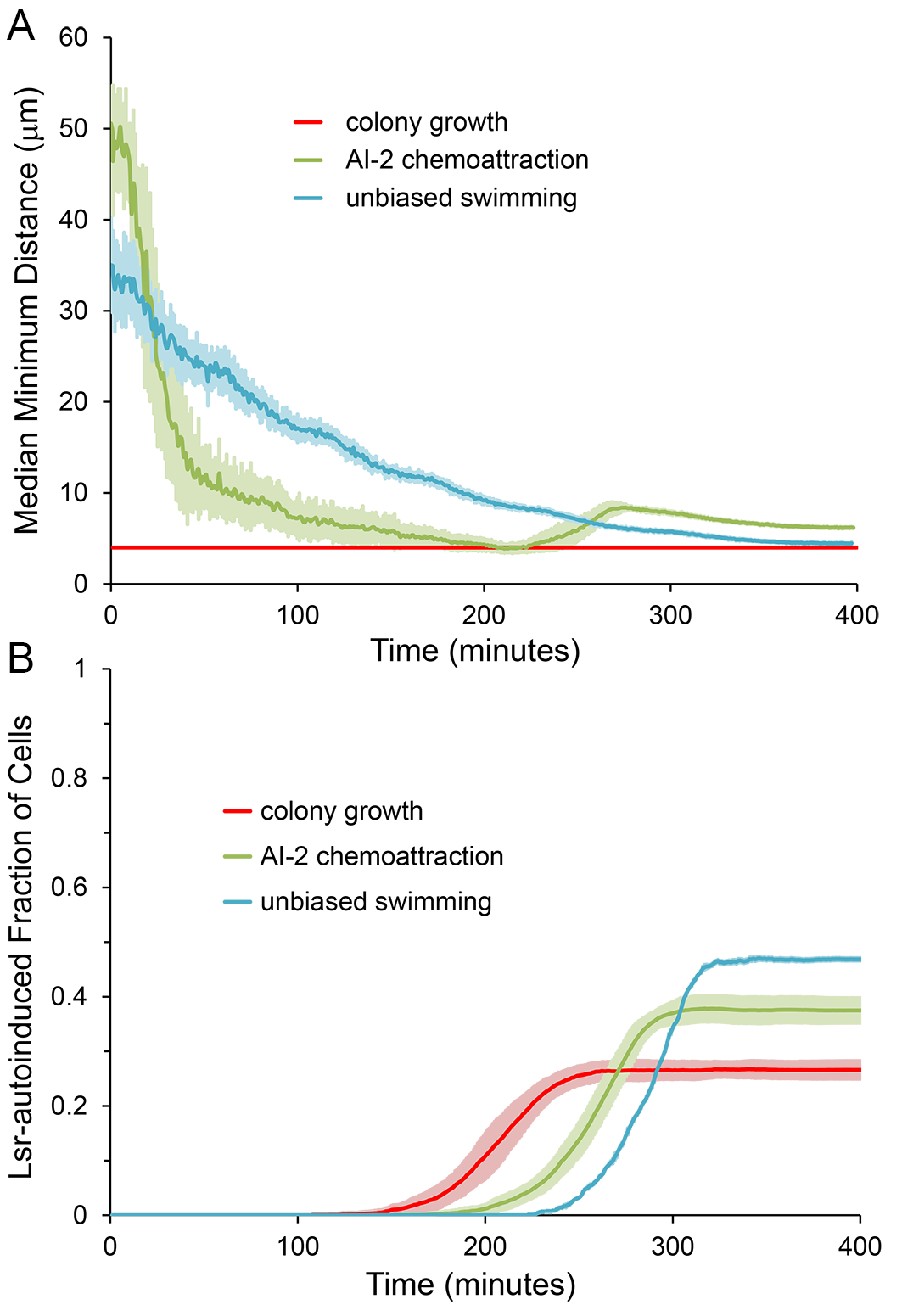

Supplement: S2 Fig — Results were derived from finite difference agent based simulations of Lsr activity. (A) The fraction of the population that was QS activated over time, in Lsr simulations of different motility, with average values set in the darker lines and the standard deviation represented by lighter surrounding shades (n = 20). (B) The median minimum cell-cell distance for populations influenced by different combinations of motility and AI-2 uptake. Dark lines are average value and the surrounding lighter shades reflect the corresponding standard deviation (n = 20). For example, cells undergoing colony growth had a predefined, regular distance between them, thus a single value prevailed across the entire time course and variability was zero. (TIF) [file pcbi.1004781.s002.tif]

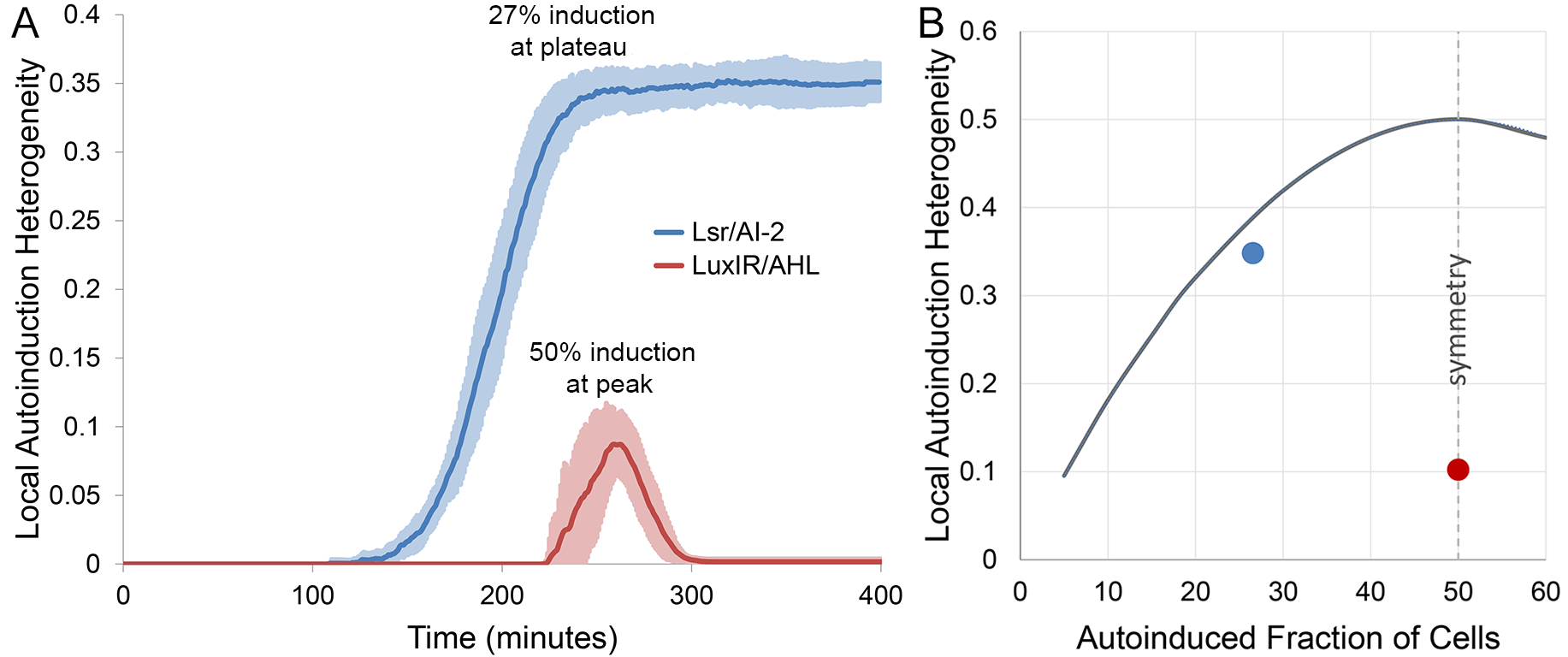

Supplement: S3 Fig — Results were derived from finite difference agent based simulations of LuxIR activity or Lsr activity using a median basal of 487.8 and a coefficient of variance of 0.052 (σ = 0.0225) for bacteria growing in a colony. (A) The dark lines represent the average local heterogeneity of 20 simulations, while the lighter, surrounding shades represent the standard deviation of those values. Also noted are the percentage of QS activity at the plateau of heterogeneity for Lsr simulation (represented in blue) and the percentage QS activity at the peak of heterogeneity for LuxIR simulations (represented in red). This is relevant, since the measure of local heterogeneity used is sensitive to the fraction of QS activation. This is seen in (B) for measures of colonies wherein QS state was assigned for each cell with a probability reflecting the percent QS activation. For enhanced context, the blue dot represents the heterogeneity for Lsr at plateau, whereas the red dot represents the heterogeneity for LuxIR at 50% activation. (TIF) [file pcbi.1004781.s003.tif]

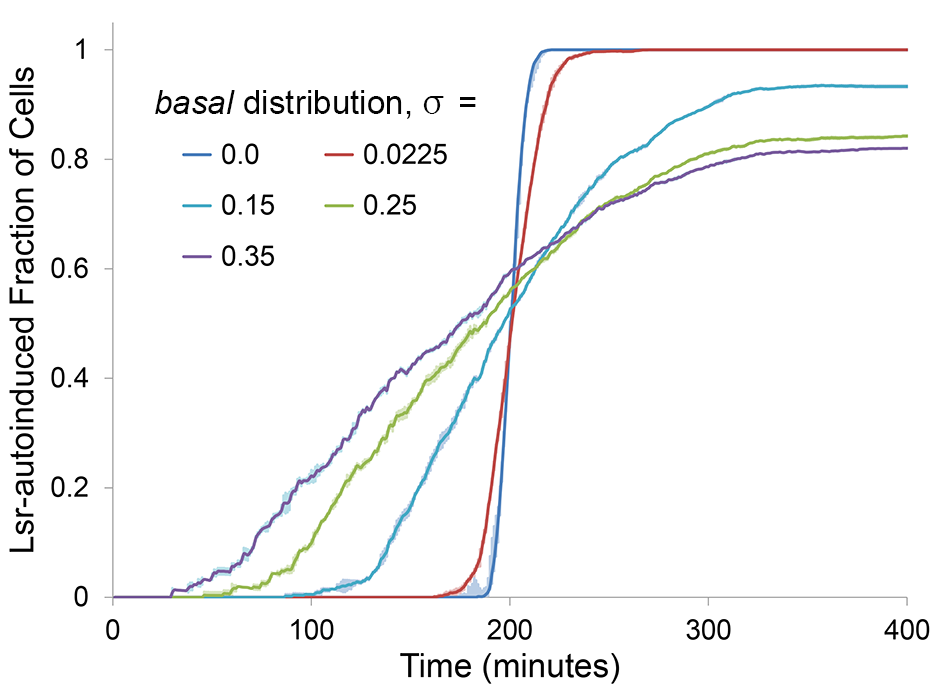

Supplement: S4 Fig — Results were drawn from finite difference agent based simulations of Lsr activity using a reduced rate of induced AI-2 import. The fraction of the population that was QS activated over time given changes to the variation for the distribution of the basal rate of AI-2 import. Variation was shifted over a range from 0.0 to 0.35. (TIF) [file pcbi.1004781.s004.tif]

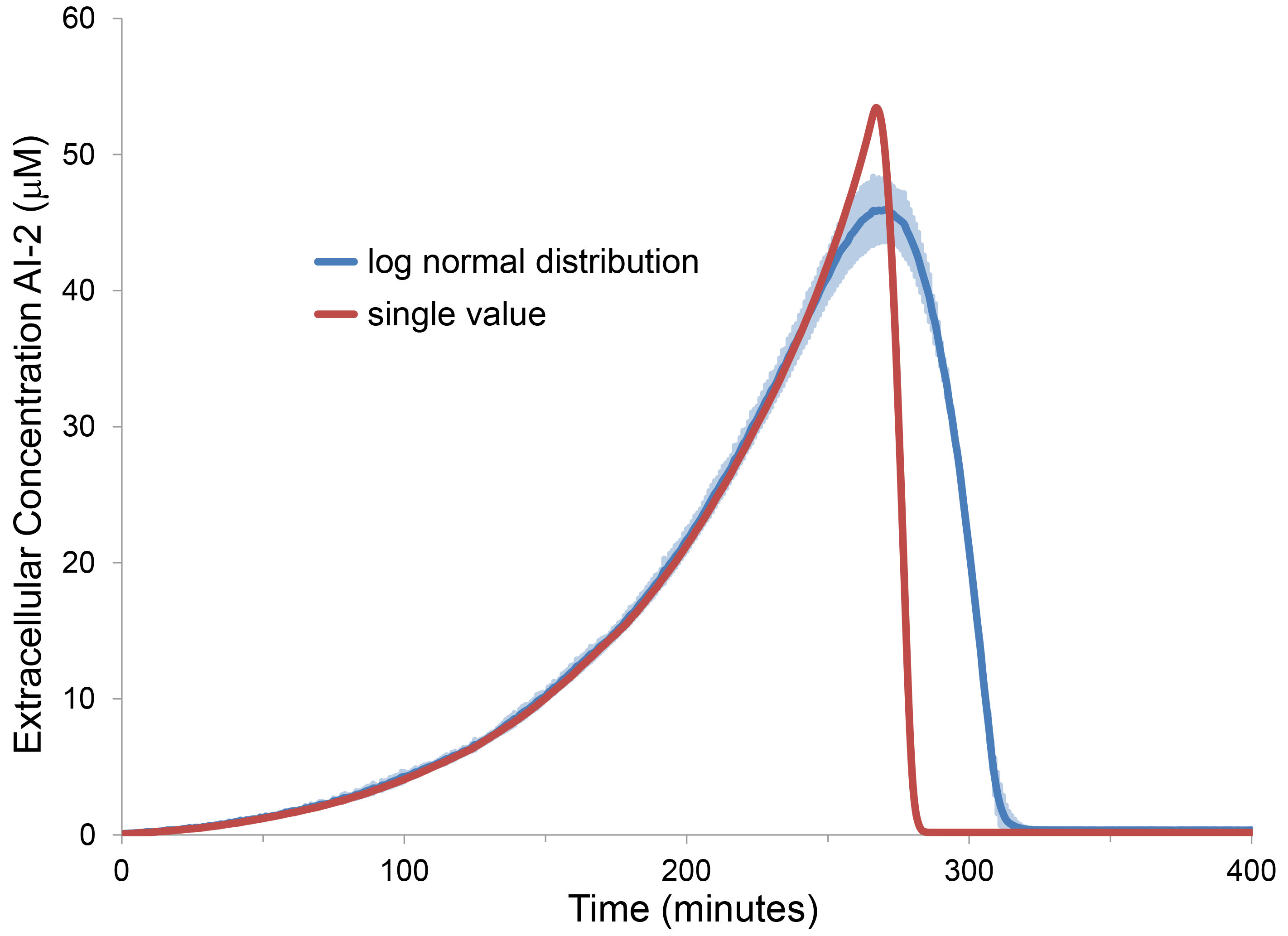

Supplement: S5 Fig — Juxtaposition of the solution for extracellular AI-2 for a simulation of cells with a single basal value versus the average solution of extracellular AI-2 for a simulation of cells with a log normal distribution of the parameter basal (TIF) [file pcbi.1004781.s005.tif]

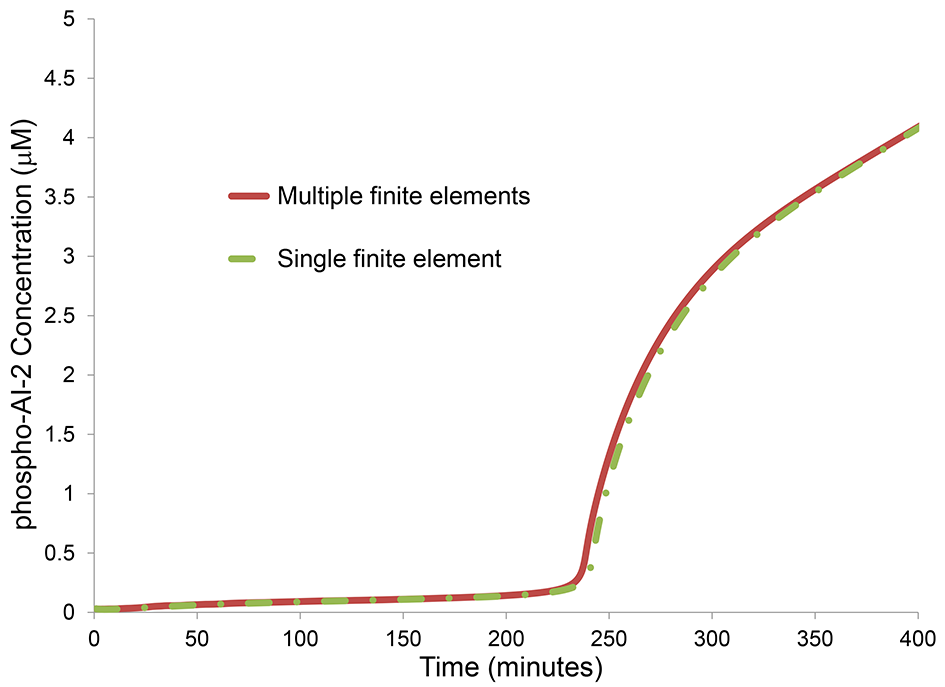

Supplement: S6 Fig — The average trajectory of AI2-P for cells with the same parameter sets in simulations where the environment was defined as either a single finite difference element or by the standard array of elements as defined in the methods. Modeling with a single finite difference element eliminates spatial noise as a source of difference between cells. The addition of noise through the full implementation of finite difference elements, adds spatially associated noise to the simulation. This did not result in a significant change in the average trajectory of AI2-P. (TIF) [file pcbi.1004781.s006.tif]

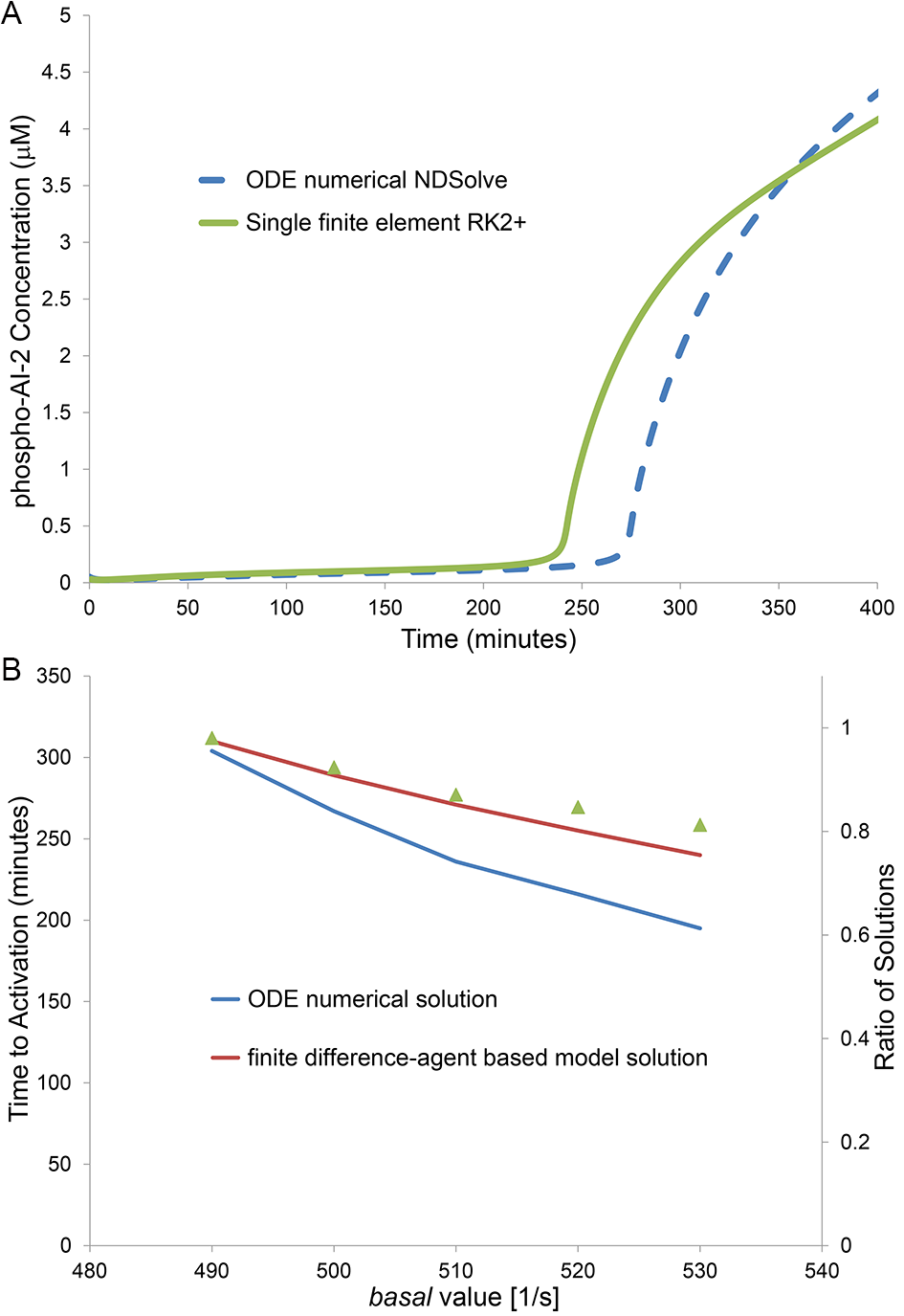

Supplement: S7 Fig — A AI2-P trajectory from implicit numerical methods and the average AI2-P concentration from the finite difference-agent based approach. Here, cells from the finite-difference-agent based solution all held the same parameter values as that from the pure ODE solution. In the pure ODE approach, cells were modeled as a dependent variable. Ideally, the two solutions would bear identical traces. B The rate to activation was assessed by fitting the function, f(t), from 12–152 minutes to a first order linear regression, g(t). The first time point at which f(t)-g(t)>2g(t) was considered the point of activation. The time to activation for each value of basal was calculated and the bearing on the solution by the modeling and numerical method used was evaluated by direct comparison along the primary axis and according to the ratio of activation times for the finite difference-agent based solution to the pure ODE solution on the secondary axis. (TIF) [file pcbi.1004781.s007.tif]
