# Supplementary material for: Quorum Sensing Desynchronization Leads to Bimodality and Patterned Behaviors
Source: PLoS Comput Biol. 2016 Apr 12;12(4):e1004781. doi: 10.1371/journal.pcbi.1004781 (PMC4829230; doi:10.1371/journal.pcbi.1004781)
Supplement: S1 Table — (DOCX) [file pcbi.1004781.s009.docx]

**S1 Table. Estimated parameter values**

| **Parameter** | **Value** | **Parameter** | **Value** | **Parameter** | **Value** |
| --- | --- | --- | --- | --- | --- |
| 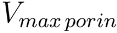 | 8000 μM/min | 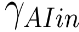 | 0.15 /min | 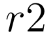 | 7x10^-7^ μM^4^ |
| 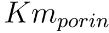 | 1 μM | 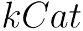 | 50 /min | 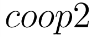 | 4 |
| 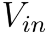 | 2x10^12^ /(min*μM^3^) | 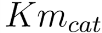 | 1.4 μM | 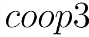 | 6 |
| 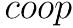 | 4 | 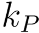 | 18.8 /min | 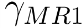 | 0.693 /min |
| 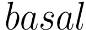 | 500 μM/min | 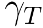 | 0.01 /min | 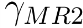 | 0.693 /min |
| 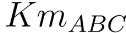 | 0.5 μM | 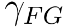 | 0.01 /min | 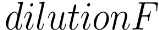 | 10^-12^ |
| 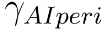 | 0.015 /min | 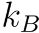 | 18.8 /min | 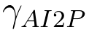 | 0.3 /min |
| 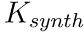 | 220 μM/min | 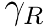 | 0.01 /min | 2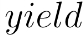 | 0.032 /min |
| 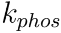 | 80 /min | 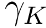 | 0.01 /min | 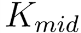 | 2250 μM |
| 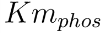 | 1.4 μM | 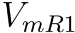 | 2x10^-5^ μM/min | 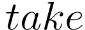 | 4.5x10^-7^ [Substr]/[cell] |
| 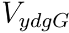 | 1250 μM/min | 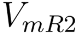 | 2x10^-5^ μM/min |  |  |
| 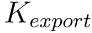 | 0.5 μM | 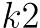 | 5x10^6^ |  |  |
