# Supplementary material for: Quorum Sensing Desynchronization Leads to Bimodality and Patterned Behaviors
Source: PLoS Comput Biol. 2016 Apr 12;12(4):e1004781. doi: 10.1371/journal.pcbi.1004781 (PMC4829230; doi:10.1371/journal.pcbi.1004781)
Supplement: S1 Text — (DOCX) [file pcbi.1004781.s010.docx]

**Supporting Informatio**

# Supporting Methods

## **Lsr ODE Model**

Generalities and scope. The model described herein was developed to simulate Lsr system behavior for bacteria in a batch reactor between lag and early stationary growth phases. At the endpoint, AI-2 should be functionally depleted from the supernatant [S1] and [26, 27] from main, with extracellular concentrations peaking between 4-6 hours [S2] and [27] from main. The developed equations relied on Hill and Michaelis-Menten like expressions to encapsulate reaction rate behaviors. In order to investigate population bifurcation, separate sets of ODEs were run simultaneously, modeling multiple cells sharing the same extracellular space.

Lsr system components were modeled as translated from polycistronic mRNA species *lsrRK* and *lsrACDBFG*, the transcription of which was considered a function of LsrR and intracellular AI-2 concentrations. The proteins LsrK and LsrR were modeled as distinct species. LsrA, LsrC, LsrD, and LsrB were folded into a single type of entity, OP, as they form a complex ABC type importer. Similarly, LsrF and LsrG were modeled as “LsrFG”, for both enzymes use the same substrate (AI2-P) and neither’s products are known to feed back onto Lsr activity. That is, LsrF and LsrG were functionally equivalent for our specific purposes [S1]. Of the proteins outside the divergent Lsr operons that influence Lsr activity in *E. coli* (*luxS*, *ydgG*, *crp*, and *pts*), none are themselves modulated by Lsr activity [S1] and [34,35] from main. Moreover, the concentration of these species have not been shown to markedly vary within the time frames of interest. These proteins were therefore considered constant and their activity rates were simplified to a Michaelis-Menten like dependence on the substrate alone.

mRNA expression. Production of mRNA species was modeled with a modified Hill equation derived from an expression for the fraction of free DNA to total DNA (free DNA + repressed DNA) as a function of LsrR and AI2P concentrations. Whereas active LsrR was modeled as a tetramer [S3], AI2P cooperativity was asserted. Cooperativity for AI2P derepression was assumed to be on the order of cooperativity for protein/DNA binding. A lower bound for such cooperativity might be 1.38-2.72, which was measured for a nonspecific interaction between DNA and a multidomain protein in *Mycobacterium smegmatis* [S4]*.* An upper bound for such interactions may be around 10 [S5]. As each LsrR has a distinct AI2P binding domain, we chose 4 as a baseline level of cooperativity. The trajectory of the system using this cooperativity was similar to that for degrees of cooperativity greater than 4. At tested cooperativities less than 4, the drop in activation ‘sharpness’ was sufficient to noticeably dull the rate of AI-2 recompartmentalization from the extracellular space. The transcription rates for the two different polycistronic mRNA species were set as equivalent, as the data on the relative strength of expression toward LsrA and LsrR is inconsistent [S6] and [32] from main.

Protein synthesis. Lsr proteins expressed from the same polycistronic mRNA species were modeled as having the same translation rate. While different ribosome binding sites along the polycistrons are likely to have different affinities for ribosomes, in the absence of data, the co-regulation implied by operon structure and protein function was given overriding consideration.

mRNA and protein degradation. As an additional simplification, both polycistronic mRNA species were modeled with the same rate of degradation, with a half-life on the order of 10 minutes, which was an order of magnitude faster than that used for proteins. While Lsr proteins are expected to have different vulnerabilities to proteolytic degradation, the result is either higher or lower quasi-steady state levels. The effects of such differences could be accounted for by either lowering or increasing corresponding unconstrained enzymatic rates.

Cell growth. Cell growth was modeled as Monod growth. The maximum rate and Monod saturation rate constants of 0.032/min and 75 μM, respectively, were had from a fit to OD_600_ measurements from a batch growth at 37^o^C. The growth rate also informed the dilution of cellular components. As a generality, the rate of cell growth strongly influenced simulation results, as increasing cell density accelerated extracellular AI-2 accumulation. That is, faster growth decreased the time to autoinduction.

AI-2 transport. Instantaneous concentration and dilution associated with transport between extracellular and intracellular spaces was treated by including a dilution/concentration term of 10^12^ as seen in equation [32] from main. This dilution factor accounted for the difference between the femtoliter intracellular environment and the milliliter term associated with cell concentration.

Influx into the periplasm through porins was modeled as a diffusion process with a Michaelis-Menten form, rate limited at high concentrations, but otherwise proportional to the concentration difference between extracellular and periplasmic compartments. Influx through Lsr ABC type complexes was modeled as a function of importer complex concentration and periplasmic AI-2. The transporter’s component proteins were treated as a single species. Transporter complex was assumed to form with a cooperativity of 4, as a reflection of the fact that four independent components are involved in complex formation, namely LsrA, LsrB, LsrC, and LsrD. Although the nucleotide binding component, LsrA, operates as a dimer, any additional degree of freedom was considered to be eliminated by the fact that the LsrA dimer pair is fused together. Further, this transporter complex was asserted to act upon periplasmic AI-2 according to a Michaelis-Menten like dynamic. Michaelis-Menten like dynamics were also asserted for the operation of the low flux importer (possibly rbs or the PTS system) as well as for AI-2 export through YdgG. That is, these processes were assumed to be constrained by a maximal velocity above a saturating AI-2 concentration, and that at lower AI-2 concentrations activity was approximately linear with respect to AI-2 itself.

AI-2 degradation and synthesis. The rate of extracellular AI-2 degradation was assumed to be minimal based on a lack of attenuated AI-2 activity in bioassays after incubation of *in vitro* synthesized samples overnight at 37^o^C. The rate of cytoplasmic AI-2 degradation was set significantly higher to account for experiments measuring cytoplasmic AI-2 in *E. coli* without functional Lsr activity, wherein cytoplasmic AI-2 concentrations dropped significantly once the stationary phase had been well established [27] from main. While the time frame of this marked decrease fell outside the scope of *in silico* experiments herein, it nonetheless suggests the existence of mechanisms that degrade AI-2 intracellularly, independent of Lsr system expression. Periplasmic degradation of AI-2 was modeled as intermediate to the extracellular and cytosolic rates, and did not bear strongly on the activity of the Lsr system, since the absolute moles in periplasmic pools were limited compared to extracellular and intracellular species.

The same experiments that provide evidence for this degradation also suggest that the rate of synthesis is not constant [27] from main. As the expression of *luxS* and *pfs* are not strongly varied across time [32] from main, decreased AI-2 production may be a result of a slower activated methyl cycle. Nonetheless, we model AI-2 synthesis as constant, which over the course of the time scale of interest we assume to be operationally approximate.

### Equations

The specific form of the equations used and the parameters values used herein were as follows:

1. $\begin{matrix} \frac{d [AI_{peri}]}{dt}=V_{\max porin}\frac{[AI_{out}]-[AI_{peri}]}{Km_{porin}+ [AI_{out}]+[AI_{peri}]}- \\ (V_{in}[OP]^{coop}+basal)\frac{[AI_{peri}]}{Km_{ABC}+[AI_{peri}]}-(\gamma_{AIperi}+\mu(t))[AI_{peri}] \end{matrix}$ ,
2. $\begin{matrix} \frac{d [AI_{in}]}{dt}=K_{synth}+(V_{in}[OP]^{ coop}+basal)\frac{[AI_{peri}]}{Km_{ABC}+[AI_{peri}]}- \\ k_{Phos}[LsrK]\frac{[AI_{in}]}{Km_{Phos}+[AI_{in}]}-V_{ydgG}\frac{[AI_{in}]}{K_{export}+[AI_{in}]}-(\gamma_{AIin}+\mu(t))[AI_{in}] \end{matrix}$,
3. $\begin{matrix} \frac{d [AI2P]}{dt}=k_{Phos}[LsrK]\frac{[AI_{in}]}{Km_{Phos}+[AI_{in}]}- \\ k_{Cat}[LsrFG]\frac{[AI2P]}{Km_{Cat}+[AI2P]}-(\gamma_{AI2P}+\mu(t))[AI2P] \end{matrix}$,
4. $\frac{d [OP]}{dt}=k_{P}[mRNA2]-(\gamma_{T}-\mu(t))[OP]$,
5. $\frac{d [LsrFG]}{dt}=k_{P} [mRNA2]-(\gamma_{FG}-\mu(t))[LsrFG]$,
6. $\frac{d [LsrR]}{dt}=k_{B} [mRNA1]-(\gamma_{R}-\mu(t))[LsrR]$,
7. $\frac{d [LsrK]}{dt}=k_{B} [mRNA1]-(\gamma_{K}-\mu(t))[LsrK]$,
8. ,
9. $\frac{d [mRNA2]}{dt}=V_{mR2}\frac{1}{1+\frac{k_{2}[LsrR]^{coop2}}{r_{2}+[AI2P]^{coop3}}}-(\gamma_{MR2}+\mu(t))[mRNA2]$,
10. $\begin{matrix} \frac{d [AI_{out}]}{dt}=dilutionF [cells](V_{max porin}\frac{[AI_{peri}]-[AI_{out}]}{Km_{porin}+[AI_{out}]+[AI_{peri}]}- \\ V_{ydgG}\frac{[AI_{in}]}{K_{export}+[AI_{in}]}) \end{matrix}$,
11. $\frac{d [cells]}{dt}=[cells]\frac{yield [Substr]}{K_{mid}+[Substr]}$,

$$\frac{d [Substr]}{dt}=-take [cells]\frac{yield [Substr]}{K_{mid}+[Substr]}$$

, where [*Substr*] represents the concentration of substrate, [*AI_peri_*] represents the concentration of periplasmic AI-2, [*AI_in_*] represents the concentration of cytoplasmic AI-2, [*AI_out_*] represents the concentration of extracellular AI-2, [*AI2P*] represents phosphorylated AI-2, [*LsrR*] represents LsrR concentration, [*LsrK*] represents LsrK concentration, [*LsrFG*] represents LsrF and LsrG concentration, [*mRNA2*] represents *lsrRK* concentration, [*mRNA1*] represents *lsrACDBFG* concentration, and [*OP*] represents transporter protein concentration.

Parameter Values. A table of parameter values can be found in **S1 Table**. The maximal rate of transport through the alternative importer, as governed by *basal*, was set lower than the rate of export through YdgG, allowing extracellular AI-2 accumulation at baseline levels of Lsr activity. The remaining parameters were set such that this initial AI-2 flux out was overcome by Lsr activation.

Specifically, according to BB170 bioassays of culture supernatant, AI-2 appears to peak between 4-5 hours in a batch culture. FRET-LuxP assays and bioassays suggest that the peak concentration is less than 80 μM and greater than 40 μM, respectively [S2] and [27] from main. Once extracellular accumulation stalls, AI-2 appears to be drawn down to low concentrations of AI-2 in less than an hour and then to concentrations below bioassay sensitivity within the next hour.

Asserting that transcription is directly coupled to translation for LacZ, transcription was fit to Miller assays of LacZ expression from pLsrA and pLsrR promoters, indicating that transcription begins evolving prior to 4 hours after culture initiation, reaching a several fold higher level of expression prior to 6 hours [32] from main.

While the selected parameter set in combination with the given ODEs produced a much faster depletion of extracellular AI-2 than was observed experimentally, the fraction of QS induced cells evolved over an extended period of time [13] from main, and it was this evolving population’s averages to which the model was fit (**S5 Fig**). In order to generate an evolving fractional Lsr autoinduction, a tractable number of cells was modeled within a finite difference agent based scheme. Each cell’s processes were modeled by its own set of ODEs and the cell population was slightly desynchronized in order to generate the fractional induction seen by Tsao *et al* [13] from main.

A parameter search was carried out to identify parameter sets satisfying the above criteria. Among other measures of fit, the time to Lsr autoinduction was sensitive to changes in all parameters effecting transport due to their direct bearing upon the balance of accumulating cytoplasmic AI-2 and corresponding AI2-P species. The parameter space satisfying the available data including extracellular AI-2 concentration, was nonetheless broad with similar sensitivities and behaviors over a range of values.

Initial values. Initial values were set according to approximate steady state values for a system without lacking AI-2 production, where the Lsr system was uninduced: [cells] = 3e7, [*Substr*] = 1500 μM, [*AI_peri_*] = 0.015 μM, [*AI_in_*] = 0.2 μM, [*AI_out_*] = 0.02 μM, [*AI2P*] = 0.03 μM, [*LsrR*] = 0.0012 μM, [*LsrK*] = 0.0012 μM, [*LsrFG*] = 0.0012 μM, [*OP*] = 0.0012 μM, and [*mRNA2*] = [*mRNA1*] = 0.

Numerical solutions. The equations and parameters were solved using NDSolve in Mathematica 8.0 utilizing “StiffnessSwitching” methods. The solution required the “StiffnessSwitching” option, without which, the solution was unstable.

## **Finite difference-agent based model**

Modeled environment. The environment was defined as a 500 x 500 x 6 μm volume, and was divided into 2 x 2 x 6 μm elements. Cells either exported or imported AI-2 from their intracellular space into or from the finite difference element in which their cell centers were found. AI-2 also diffused between finite difference elements as modeled by a forward in time-central in space scheme with an estimates diffusion coefficient of 5x10^-7^ cm^2^/s. The boundaries of the simulation were modeled as impermeable.

Adaptation of equations and solutions. With few exceptions, the previously described ODEs were repurposed without modification in the finite difference/agent based model. Growth was one exception, as cell division was treated a discontinuous stochastic event. Monod growth dynamics informed the median of a log normal distribution of doubling time with a σ of 0.05 where the median rate was updated every time step. The exchange of AI-2 between the environment and a cell was localized to the space in which the cell center was found at the beginning of the time step. This allowed a synchronized update of the final grid concentration at the end of the time step. Furthermore, the AI-2 dilution/concentration factor was adjusted from 10^12^ to 24 to account for the difference between the milliliter volume associated with cell concentration and the implied volume of grid elements.

Cell ODE numerical solution method. The numerical method used in the agent based modeling was a fourth order Runga-Kutta with an *ad hoc* allowance for stiffness. In order to achieve efficient calculation, we used an explicit method with the exception of periplasmic AI-2 after transporter concentration exceeded 5 times its initial concentration. This threshold was chosen based upon NDSolve interpolation, as a point at which the Lsr system including transporter expression and subsequent concentration had already begun its transition to an active state. After this threshold was surpassed, porins were assumed to be the rate limiting element in the transport of AI-2 from the extracellular space to the cytosol, and modeling was made to reflect this by substituting terms describing ABC-type transporter activity with terms describing porin activity; furthermore, periplasmic AI-2 was held at zero, reflecting the numerical solution from the ODE system described in previously.

Cell division. Cell division was governed by individual counters that incremented at each time step. Once a cell’s counter exceeded its doubling time, division occurred. At the time zero, cell counters were set randomly between zero and the maximum allowed value. Doubling time was varied between cells according to a mean growth rate based on Monod kinetics (with parameters found in **S1 Table**) with a log normal distribution (variance of 0.05) in order to desynchronize cell doubling. Upon division, both mother and daughter cells acquired new growth rates from a log normal distribution, while bearing duplicate properties including initial position, and their age counters were reset to zero. Newly initialized cells were assigned a basal rate of AI-2 flux through alternative importer pathways (the parameter, *basal*) or a rate of AHL synthesis, for Lsr or LuxIR simulations respectively.

AI-2 diffusion. AI-2 diffusion was modeled using a central difference approximation. While both truncation and roundoff error arise from this process, the overall behavior of the simulation was not expected to be dramatically impacted, as clustering behavior also obtained when elements were four times the size. The diffusion coefficient used (~5x10^-7^ cm^2^/s) was approximated using the Wilke-Chang correlation [S7].

Time interval ordering. The order of calculations within a time step was as follows. First, the average growth rate was determined from a Monod growth dynamic solved by a fourth order Runga-Kutta method as a function of substrate concentration and *E. coli* density. Second, each *E. coli* divided or did not divide, marginally accommodated its AI-2 chemotactic threshold (if appropriate), and moved (according to the particular scheme employed). For the specific purposes of the simulations reported here, cells moved randomly in space each time step at an average rate of 20 μm/sec, coming to an average distance of 1.3 μm per time step. Correspondingly, cells rarely moved more than one grid element at a time for any given time step. Finally, each grid was subject to calculations approximating diffusion. That is, AI-2 was allowed to move according to molecular diffusivity and distance/time calculations. After all diffusion processes were calculated over the entire environment, the exchange of AI-2 between bacteria and the grids in which the bacteria were present was calculated according to Lsr dynamics governed by previously described ODEs. The sum of these changes to AI-2 concentration were then applied to each grid and each cell. Time step and grid size were chosen such that moderate changes to these measures resulted in qualitatively indistinct outcomes.

All random assignments were drawn according to the Mersenne-Twister algorithm [S8]. Seeds for the algorithm were changed every simulation.

# Supporting Results

**System sensitivity to basal rate of AI-2 uptake.** As expected, system behavior was sensitive to parameters that markedly affected the rate of AI2-P accumulation when Lsr system expression was low (**S1 Fig**). Among these was the parameter *basal* (**S1E Fig**). With increasing *basal* value, the rate of AI2-P accretion increased, accelerating the time to Lsr activation. Specifically, an ~8% increase in the rate of AI-2 influx through the alternative pathway from the base value resulted in a ~21% reduction in the time to activation.

**
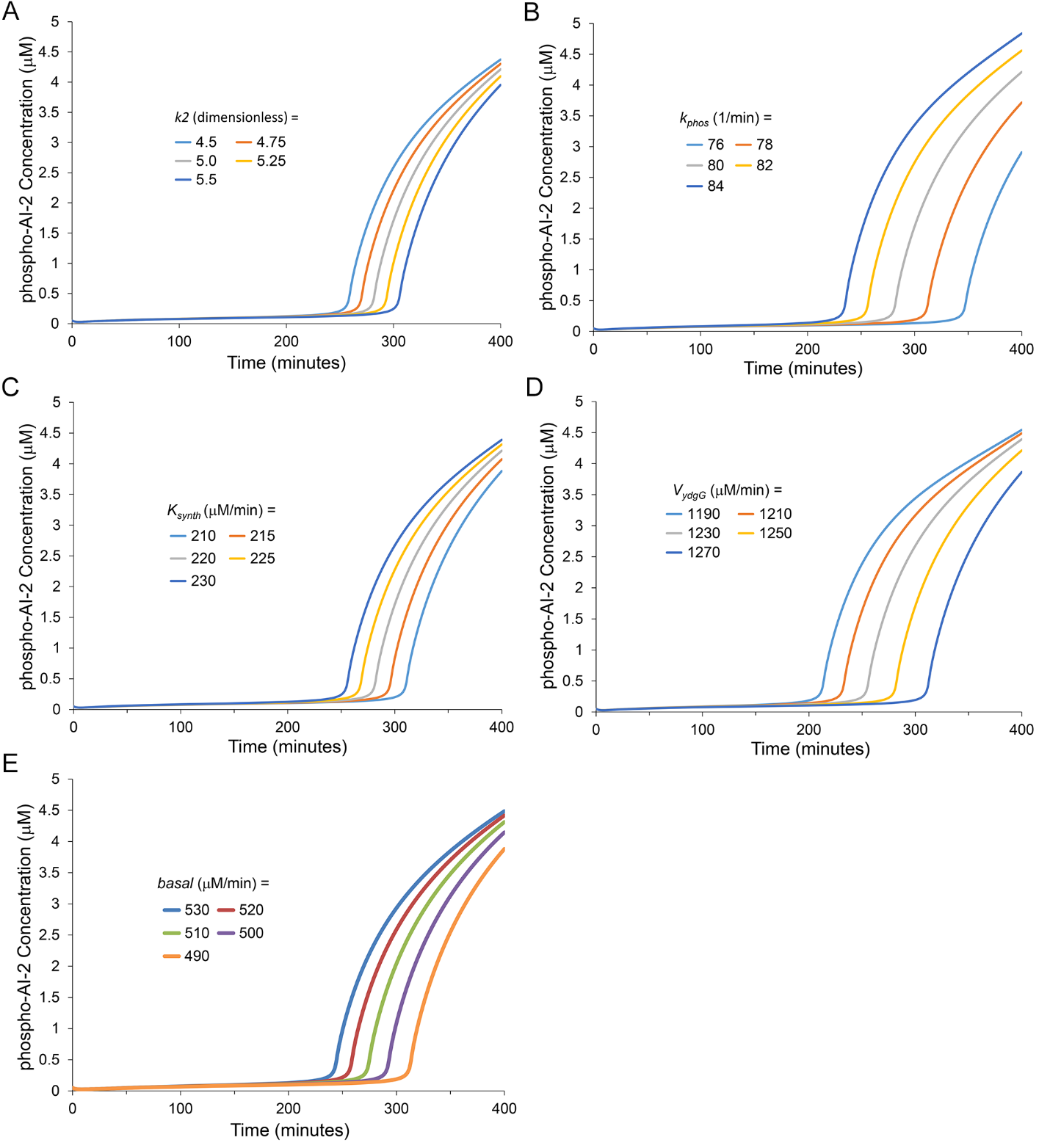
**

**S1 Fig. System sensitivity to parameter changes that manifested in shifting time to Lsr autoinduction.** Solutions to ODE’s modeling Lsr activity, where rapidly increasing phosphorylated AI-2 concentration indicates Lsr autoinduction. Shifts in the time to activation were associated with reported parameter value changes. Changes to *k2* (transcription Hill parameter; A), *k_phos_* (phosphorylation; B), *K_synth_* (AI-2 synthesis; C), *V_ydgG_* (AI-2 export; D), and *basal* (low affinity AI-2 import; E) are presented.

**Feedback from motility modes on fraction Lsr activation related to intercellular distance.** The cell-cell distance changed as a function of the motility mode being tested. This included an AI-2 chemoattracted motility not previously discussed that produced a cell-cell distance between undirected swimming and colony growth (**S2A Fig**). As seen in **S2B Fig**, among the three simulated motility modes, non-taxis swimming populations were the slowest to QS activate. Populations of such cells also ultimately achieved the largest proportion of stably QS activated cells. This inverse correlation persisted across motility types such that decreasing cell-cell distance (or put differently, higher density) appeared to lead to earlier activation. This dovetailed with observations in the main text connecting cell density, speed to activation, and fractional activation.

**
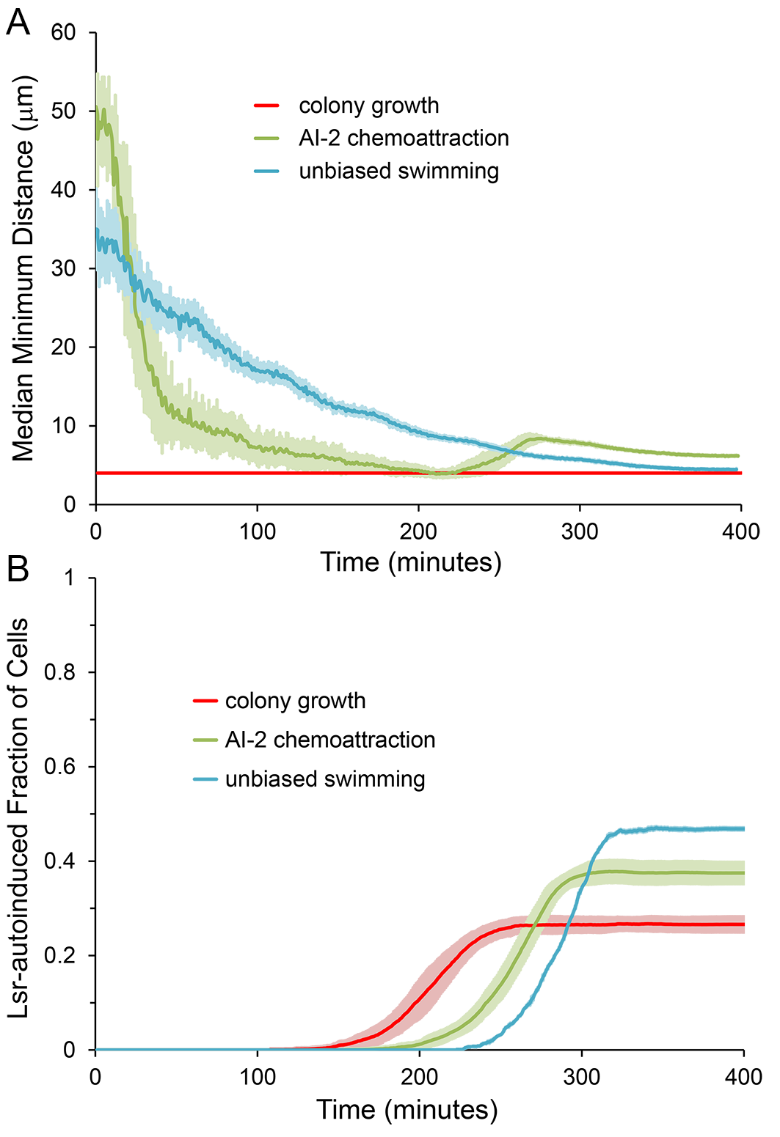
**

**S2 Fig. Measures of the difference between different modes of motility when coupled with Lsr/AI-2 dynamics.** Results were derived from finite difference agent based simulations of Lsr activity. (A) The median minimum cell-cell distance for populations influenced by different combinations of motility and AI-2 uptake. Dark lines are an average value (n = 20), while the surrounding lighter shades reflect the corresponding standard deviation. For example, cells undergoing colony growth had a predefined, regular distance between them, thus a single value prevailed across the entire time course and variability was zero. (B) The fraction of the population that was QS activated over time, in Lsr simulations of different motility, with average values (n = 20) set in a darker thinner line and a lighter surrounding shade representing the standard deviation.

**Quantifying spatial heterogeneity of LuxIR and Lsr based autoinduction in colony growth.** For further perspective on the difference between LuxIR and Lsr QS responses in colony growth (speckled versus wave front), a heuristic score representing spatial heterogeneity of autoinduction was developed. We created a scoring rubric that was highest when every cell’s neighbors, up or down, left or right (but not diagonal), were of the opposite QS state. For example, a value of one indicated a perfect checkerboard pattern of alternating QS activation. A score of zero indicated that all neighboring cells were of the same state. At each time point this heuristic score was averaged over the entire population. The values reported in **S3A Fig** represent the average trajectory of heuristic scores from twenty simulations of Lsr and LuxIR autoinduction in growing cell colonies. The average score is represented by dark blue line (Lsr) and dark red lines (LuxIR) with the surrounding more lightly shaded bands representing the standard deviation.

**
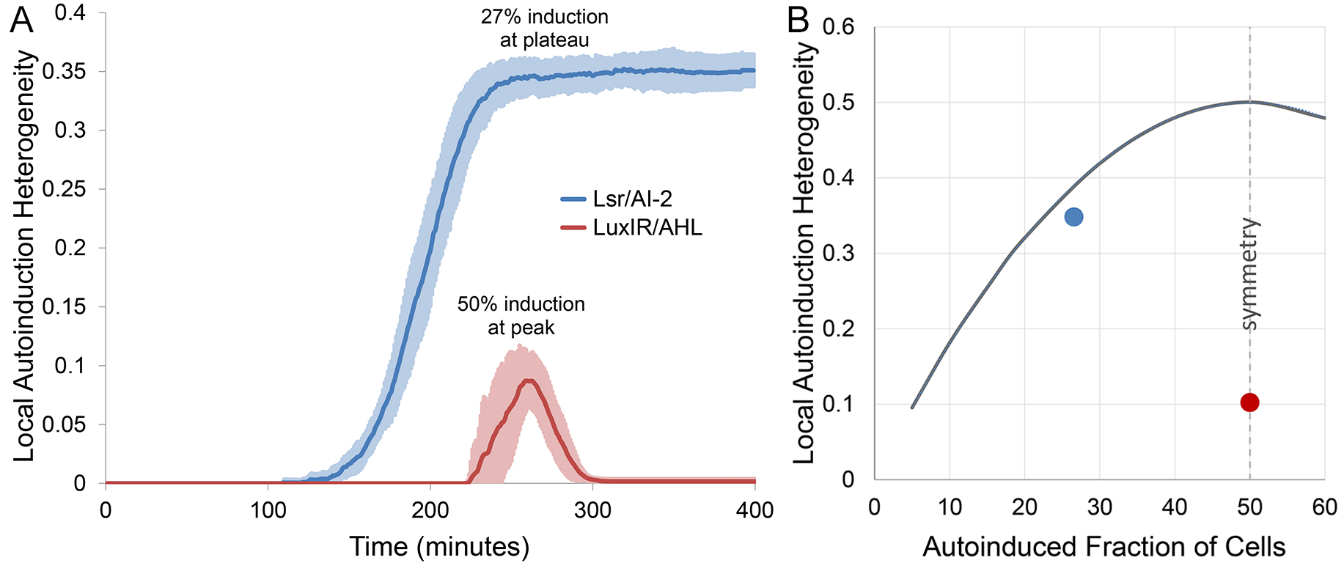
**

**S3 Fig. Local heterogeneity of Lsr versus LuxIR QS activation in colony growth.** Results were derived from finite difference agent based simulations of LuxIR activity or Lsr activity using a median *basal* of 487.8 and a coefficient of variance of 0.052 (σ = 0.0225) for bacteria growing in a colony. (A) The dark lines represent the average local heterogeneity of 20 simulations, while the lighter, surrounding shades represent the standard deviation of those values. Also noted are the percentage of QS activity at the plateau of heterogeneity for Lsr simulation (represented in blue) and the percentage QS activity at the peak of heterogeneity for LuxIR simulations (represented in red). This is relevant, since the measure of local heterogeneity used is sensitive to the fraction of QS activation. This is seen in (B) for measures of colonies wherein QS state was assigned for each cell with a probability reflecting the percent QS activation. For enhanced context, the blue dot represents the heterogeneity for Lsr at plateau, whereas the red dot represents the heterogeneity for LuxIR at 50% activation.

At time zero, all simulations began with a score of zero (QS inactive) in a colony consisting of 50 cells. For simulations of LuxIR QS, heterogeneity increased from a zero baseline as activation began near 255 minutes. LuxIR QS heterogeneity score peaked to near 0.1 when QS activation was around 50%. After this peak, the score dropped until reaching zero. This evolution reflects the pattern of QS initialization to complete colony activation—at both times, the colonies were in a consensus QS state. In Lsr/AI-2 simulations of colony growth run with a median *basal* of 487.8 and a coefficient of variance of 0.052 (σ = 0.0225), local Lsr QS heterogeneity increased from its zero baseline once activation began around 120 minutes. Heuristic scoring for Lsr activity plateaued around 200 minutes reaching a score of ~0.35.

**S3B Fig** is provided for context, representing the heterogeneity score for colonies where cells’ QS states were a function of probability alone based on the percent QS activation reported in the ordinate axis. That is, **S3B Fig** presents the heterogeneity score given randomly assigned QS activation (solid line). As the heuristic was indifferent to whether the majority of QS states are QS “on” or QS “off”, scoring was symmetric about 50% QS activation. This contextualizes the data in **S3A Fig**. Specifically it can be ascertained that the heuristic 0.1 value for LuxIR at 50% QS activation (red dot) was low relative to a colony wherein the QS state is randomly assigned. This is indicative of the “ordering” produced by LuxIR signaling. For Lsr signaling, the heterogeneity heuristic score at the 27% plateau of QS activation (blue dot) is closer to the value for a colony of completely random QS activation, comporting with the simulated Lsr signaling sensitivity to the value of *basal* which was itself randomly distributed.

#### **
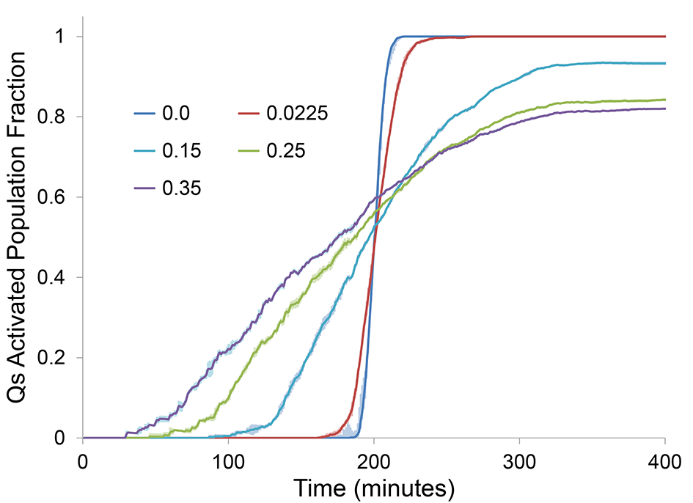
**

**S4 Fig. Slower induced import results in less negative intercellular feedback and fewer uninduced cells.** Results were drawn from finite difference agent based simulations of Lsr activity using a reduced rate of induced AI-2 import. The fraction of the population that was QS activated over time given changes to the variation for the distribution of the basal rate of AI-2 import. Variation was shifted over a range from 0.0 to 0.35.


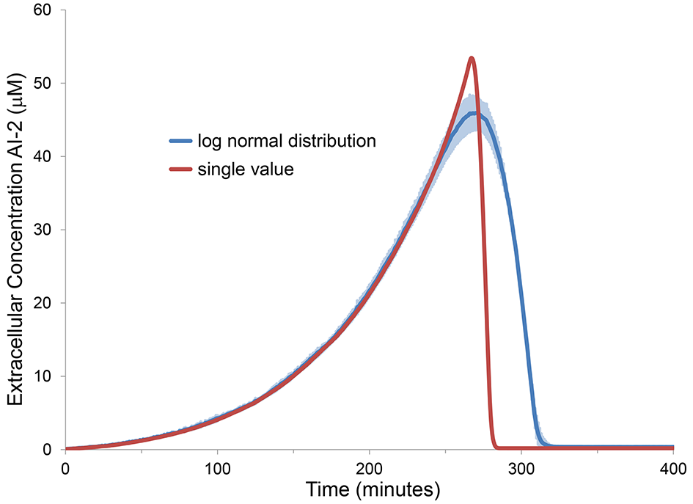


**S5 Fig. Comparison of solution for population with a single basal value versus a population with a unimodally distributed value of *basal*.** Juxtaposition of the solution for extracellular AI-2 for a simulation of cells with a single basal value versus the average solution of extracellular AI-2 for a simulation of cells with a log normal distribution of the parameter *basal*

**Minimal role of spatial heterogeneity of AI-2.** In addition to evaluating the role of heterogeneous expression at the population scale, whether spatially associated stochasticity might influence bimodal expression was also inferred, mainly from comparison of simulations using a standard finite difference scheme against simulations where the entire environment was defined by a single element. Treating the environment as homogenous made all AI-2 simultaneously available to all cells, whereas cells only interacted with AI-2 in their own element using a standard finite difference approach. As shown in **S6 Fig**, the approaches yielded highly similar AI2-P trajectories when cell motility was undirected. This was the case for all state variables modeled. Furthermore, in standard finite element environments, when governed by a single parameter set, cell populations became wholly activated over a very small window (**Fig 4B.** σ = 0). If heterogeneity arising from spatial stochasticity influenced the bimodal phenotype, population activation would be expected to be incomplete. The absence of such an effect implied that spatial stochasticity did not play a marked role in shaping bimodal response.


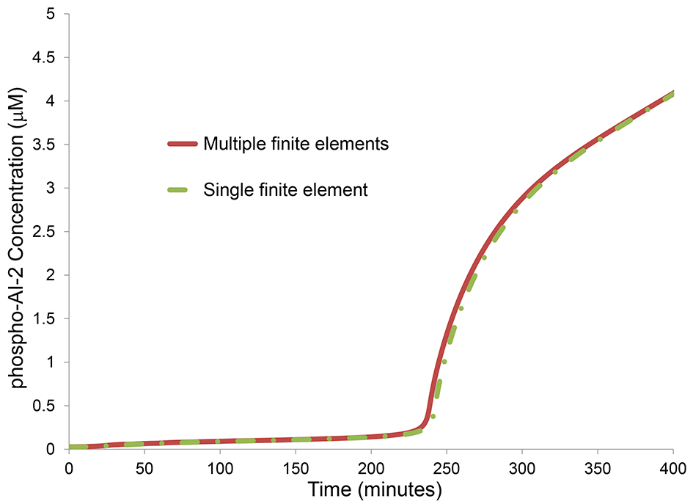


**S6 Fig. Comparison of results from single versus multiple finite difference elements to define environment.** The average trajectory of AI2-P for cells with the same parameter sets in simulations where the environment was defined as either a single finite difference element or by the standard array of elements as defined in the methods. Modeling with a single finite difference element eliminates spatial noise as a source of difference between cells. The addition of noise through the full implementation of finite difference elements, adds spatially associated noise to the simulation. This did not result in a significant change in the average trajectory of AI2-P.

**Agreement between numerical ODE and finite difference agent based solutions.** As a general comment, the agreement between numerical ODE solutions and the finite difference-agent based approach was inexact. In particular, the time to activation was offset between the two solutions as seen in **S7A Fig**. Nonetheless, the solution trajectories were similar and an evaluation of the time to activation as a function of *basal* indicated that parameter sensitivities between the solution approaches were congruous, as seen in **S7B Fig**.

**
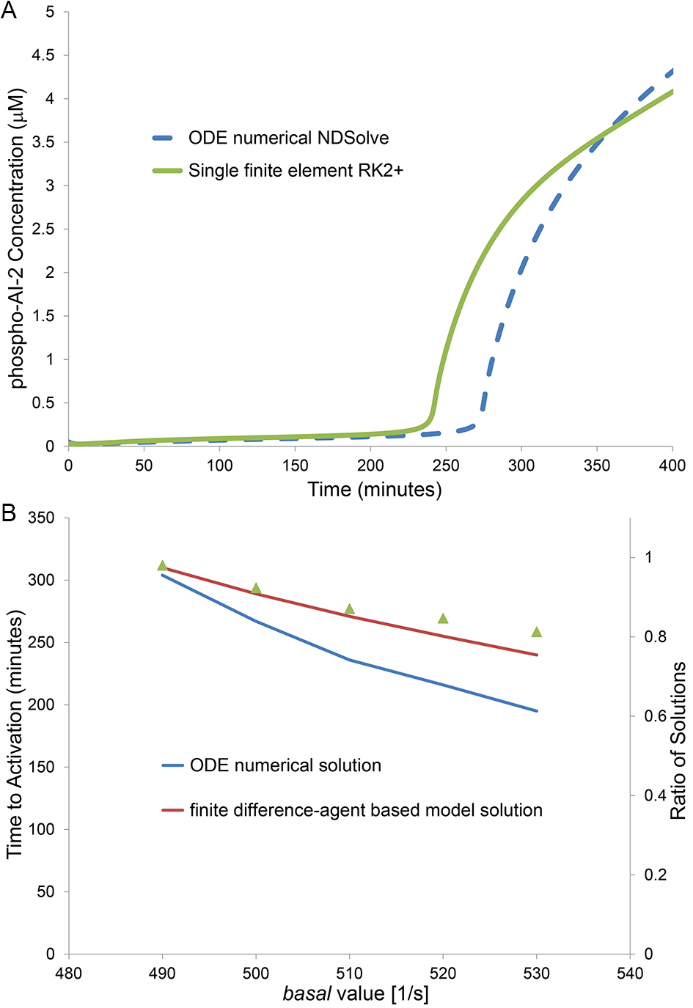
**

**S7 Fig. Congruence of solution from finite difference-agent based modeling versus implicit solution of pure ODE’s. A** AI2-P trajectory from implicit numerical methods and the average AI2-P concentration from the finite difference-agent based approach. Here, cells from the finite-difference-agent based solution all held the same parameter values as that from the pure ODE solution. In the pure ODE approach, cells were modeled as a dependent variable. Ideally, the two solutions would bear identical traces. **B** The rate to activation was assessed by fitting the function, *f(t)*, from 12-152 minutes to a first order linear regression, *g(t)*. The first time point at which *f(t)-g(t)>2g(t)* was considered the point of activation. The time to activation for each value of *basal* was calculated and the bearing on the solution by the modeling and numerical method used was evaluated by direct comparison along the primary axis and according to the ratio of activation times for the finite difference-agent based solution to the pure ODE solution on the secondary axis.

# Supporting References

**S**1. Taga ME, Miller ST, Bassler BL. Lsr‐mediated transport and processing of AI‐2 in *Salmonella* typhimurium. Mol Microbiol. 2003;50(4):1411–27.

S2. Zhu J, Pei D. A LuxP-based fluorescent sensor for bacterial autoinducer II. ACS Chem Biol. 2008;3(2):110–9.

S3. Wu H, Tsao C, Quan DN, Cheng Y, Servinsky MD, Carter KK, et al. Autonomous bacterial localization and gene expression based on nearby cell receptor density. Mol Syst Biol. 2013;9(1).

S4. Ganguly A, Rajdev P, Williams SM, Chatterji D. Nonspecific Interaction between DNA and Protein allows for Cooperativity: A Case Study with Mycobacterium DNA Binding Protein. J Phys Chem B. 2011;116(1):621–32.

S5. Von Dassow G, Meir E, Munro EM, Odell GM. The segment polarity network is a robust developmental module. Nature. 2000;406(6792):188–92.

S6. Byrd CM. Local and global gene regulation analysis of the autoinducer-2 mediated quorum sensing mechanism in *Escherichia coli*. 2011;

S7. Wilke C, Chang P. Correlation of diffusion coefficients in dilute solutions. AIChE J. 1955;1(2):264–70.

S8. Matsumoto M, Nishimura T. Mersenne twister: a 623-dimensionally equidistributed uniform pseudo-random number generator. ACM Trans Model Comput Simul TOMACS. 1998;8(1):3–30.
